# Microtubules coordinate cellularization in a coenocytic relative of animals

**DOI:** 10.26508/lsa.202603917

**Published:** 2026-10-26

**Authors:** Margarida Araújo, Marine Olivetta, Paolo Ronchi, Viola Oorschot, Arif Khan, Christian Tischer, Hiral Shah, Gautam Dey, Omaya Dudin

**Affiliations:** 1 Cell Biology and Biophysics, European Molecular Biology Laboratory (EMBL), Heidelberg, Germany; 2 Department of Biochemistry, Faculty of Sciences, University of Geneva , Geneva, Switzerland; 3 Electron Microscopy Core Facility, European Molecular Biology Laboratory (EMBL), Heidelberg, Germany; 4 European Molecular Biology Laboratory (EMBL), Data Science Centre, Heidelberg, Germany

## Abstract

Cellularization is the coordinated division of a multinucleate cell into many cells, a common life cycle transition across eukaryotes. In the best-studied model of this process, *Drosophila melanogaster*, cellularization requires actin and microtubule networks, as well as membrane vesicle delivery to drive the synchronous invagination of plasma membrane furrows. To identify unifying principles, we investigate cellularization in the ichthyosporean *Sphaeroforma arctica*, a close relative of animals with a multinucleate life cycle stage. Using live-cell imaging, ultrastructure expansion microscopy, and volume electron microscopy, we define the membrane, microtubule, and actin dynamics that accompany cellularization. Using pharmacological inhibitors and centrifugation, we show that the microtubule network positions nuclei evenly at the cortex, thereby specifying furrow positioning. Disrupting microtubules perturbed nuclear position, furrow spacing, and the efficiency of furrow ingression, leading eventually to cells of variable size and nuclear content. Our findings indicate that the microtubule network sets the spatial framework within which actomyosin and membrane delivery drive cellularization and establish *S. arctica* as a tractable model for comparative studies of this eukaryotic life cycle strategy.

## Abstract (precis)

MT networks position cellularization furrows to accurately partition multinucleate *Sphaeroforma arctica*. U-ExM, perturbations, and live imaging suggest shared patterns of cytoskeletal remodeling in eukaryotes.

## Introduction

Multinucleate cells arise through two distinct mechanisms: syncytia that form by cell fusion, or coenocytes that result from nuclear division without cytokinesis (1, 2). Some multinucleate cells persist throughout their lifetime, whereas others undergo cellularization, a specialized form of cytokinesis in which individual nuclei become encapsulated by the plasma membrane (PM) to form discrete cells (2, 3, 4, 5). Cellularization occurs across diverse eukaryotic lineages, including deep branching holozoans such as *Ichthyosporea*, chytrid fungi, and the endosperm of flowering plants, far beyond the insect embryos in which it is best studied (2, 3, 4, 5, 6, 7, 8, 9, 10, 11, 12).

In *Drosophila melanogaster*, cellularization during the 14th nuclear division transforms the multinucleate blastoderm into an epithelial monolayer (13, 14). This process depends on zygotic genome activation, nucleocytoplasmic ratio thresholds, and membrane expansion via Golgi-derived vesicles (13, 15, 16). Disruption leads to developmental defects, arrest, or lethality (3, 17, 18). Actin and microtubules (MTs) contribute in system-specific ways: in *D. melanogaster*, actin drives furrow ingression, and MTs mediate nuclear positioning and vesicle transport (9, 17, 19, 20, 21). Similarly, endosperm cellularization in *Arabidopsis thaliana* relies on radial MTs and actin cables to maintain nuclear spacing and furrow position (5, 8). In the chytrid fungus *Spizellomyces punctatus*, cellularization occurs within a spherical coenocyte with nuclei dispersed throughout the cytoplasm (4). Centrosomes migrate to the cortex, initiating vesicle-associated membrane invaginations, whereas actomyosin networks drive 3D furrow ingression to generate a polyhedral cellularization (4). Although MTs are dispensable for furrow formation, they are required for nuclear patterning and ciliogenesis (4). Thus, across multinucleate systems, uniform nuclear positioning and spacing are consistently associated with successful cellularization, emerging as a recurrent spatial constraint governing the process (4, 5, 13, 22, 23). In many multinucleate cells, microtubule-organizing centers (MTOCs) and associated MT networks set the cellular coordinate system, defining nuclear territories around neighboring nuclei and preventing clustering within the cytoplasm (4, 22, 23). Yet, how this nucleus–MT system contributes to the placement of furrows and the outcome of cellularization remains an open question.

**Figure 1.**
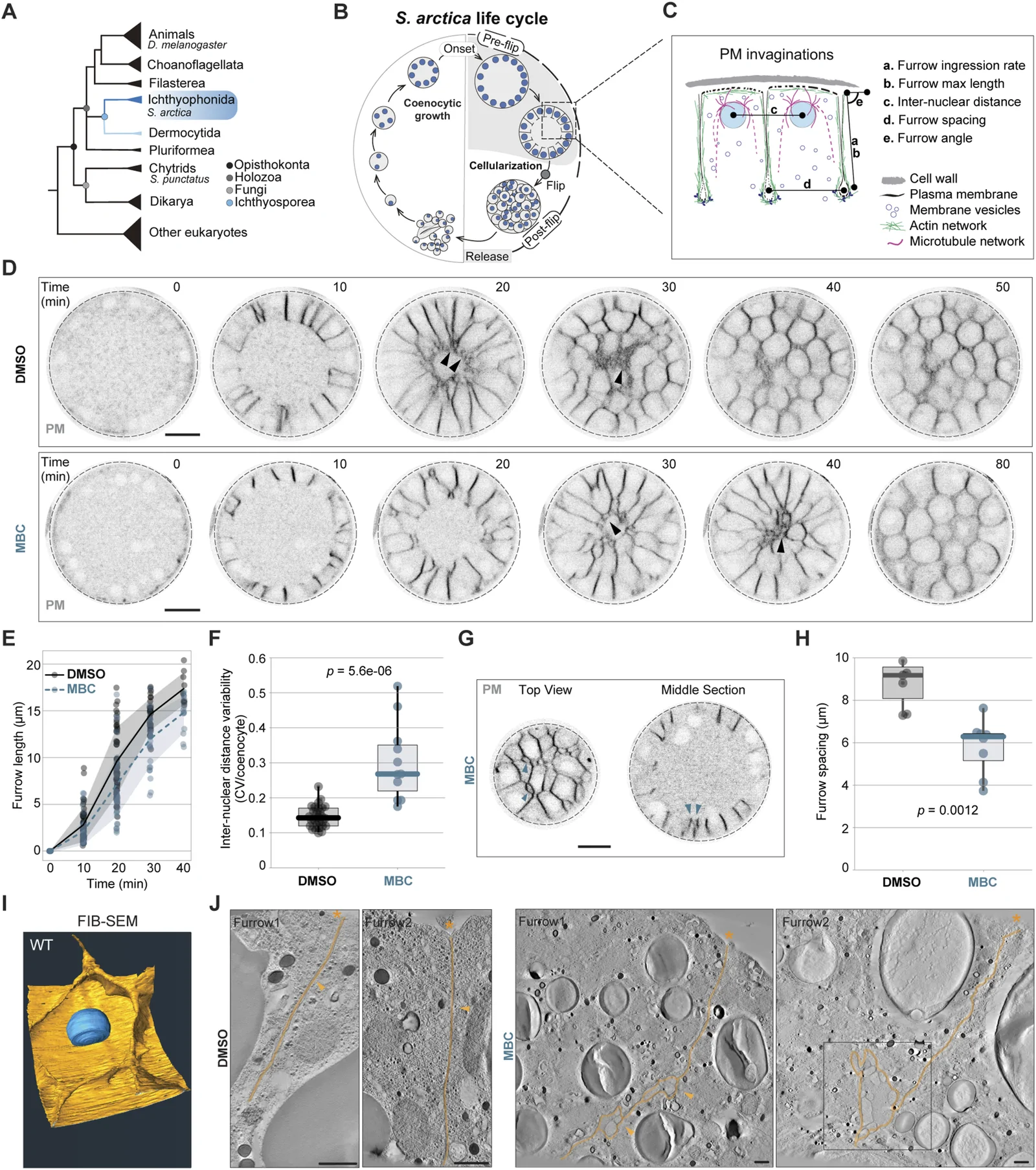
Loss of MTs alters cellularization dynamics. **(A)** Cladogram showing the position of Ichthyosporea within Holozoa and opisthokonts. **(B)** Life cycle of *S. arctica* highlighting cellularization with a gray background. **(C)** Schematic of cellularization compartments at the cell cortex. Cellular parameters measured in this study: furrow ingression rate (a), maximum furrow length (b), internuclear distance (c), furrow spacing (d), and invagination angle (e) are shown. **(D)** Time course of cellularization membrane dynamics in control and MBC-treated coenocytes labeled with FM4-64 and imaged at 10-min intervals. Arrows indicate membrane dynamics at furrow edges. Scale bar, 10 μm. **(E)** PM furrow lengths measured over time through invagination in control and MBC-treated coenocytes. **(F)** Variability of internuclear distance within a coenocyte, measured across its own nuclei from live FM4-64 imaging. Each point is one coenocyte. DMSO, 0.143 (n = 28); MBC, 0.268 (n = 11), 390 distances from 39 coenocytes, *P* = 5.6e−06. Mean internuclear distance does not differ between conditions (Fig S1G). **(G)** Top and middle section of furrow spacing in MBC-treated cells labeled with FM4-64. Scale bar, 10 μm. **(H)** Furrow spacing per coenocyte, measured peak to peak along a cortical line profile 10 min after the onset of invagination. Each point is one coenocyte. DMSO, 9.20 μm (n = 7); MBC, 6.31 μm (n = 8); 263 gaps from 15 coenocytes across four independent experiments, *P* = 0.0012. Boxes (in F, H) show median and interquartile range, and whiskers, 1.5 × IQR. Mann–Whitney U test; exact *P*-values are given. **(I)** 3D segmentation of PM (yellow) and nucleus (blue) from a FIB-SEM volume of a cellularizing coenocyte. **(J)** TEM tomography slices showing morphology of two furrows from control and MBC-treated coenocytes. Scale bar, 500 nm. Yellow outlines and arrowheads trace and indicate furrows, and yellow asterisks mark the cell surface. DMSO (n = 25 furrows from 34 tomograms, with cell surface seen in 19), MBC (n = 67 furrows from 72 tomograms, with cell surface visible in 37), total 106 tomograms imaged across different cellularization stages and conditions. Source data are available for this figure.

Among ichthyosporeans (Fig 1A), a lineage of close animal relatives, *Sphaeroforma arctica* has emerged as a tractable model for investigating mechanisms orchestrating cellularization (6, 11, 24, 25, 26, 27). *S. arctica* exhibits a life cycle in which cytokinesis is uncoupled from mitosis: cells undergo multiple rounds of nuclear division without cell division, forming multinucleate cells called coenocytes (Fig 1B) (11). Nuclei divide synchronously and, rather than occupying the interior of the coenocyte, associate with the cortex, evenly spacing out along the surface. During mitosis, *S. arctica* undergoes closed mitosis driven by a nucleus-associated acentriolar MTOC that bears similarities to the cell cycle–dependent organization of fungal spindle pole bodies (28). After coenocytic growth, *S. arctica* undergoes actomyosin-dependent cellularization through coordinated PM invaginations that progress inward from the cortex, forming a transient layer of polarized cells (Fig 1B and C) (11). Recent work has shown that *S. arctica* cellularization is regulated by the nucleocytoplasmic (N/C) ratio, with cellularization timing tightly coupled to nuclear content relative to cytoplasmic volume (26). Invaginations initiate at the cell periphery, between adjacent cortical nuclei, and advance inward, carving compartments from the cortex around each nucleus rather than assembling them from the cell interior. This developmental transition shares features with insect cellularization, including membrane furrow formation, use of conserved cytoskeletal regulators such as formins, Arp2/3, and myosin II, and transcriptional waves of cytoskeletal and adhesion genes (11, 19, 20). Once the furrows converge at the center of the coenocyte, ingression ends in an abrupt transition termed the “Flip,” where the cells of the newly closed epithelial-like layer rapidly detach from one another. Flip marks the end of cellularization and the start of the phase in which individual uninucleate cells prepare for release.

The synchronous partitioning of the coenocyte into uniform distinct cellular compartments at cellularization would require accurate nuclear and furrow positioning and membrane delivery to drive consistent ingression dynamics, and yet, how all these aspects are regulated and coordinated with cytoskeletal reorganization remains unknown beyond insect models. Here, we address the factors contributing to the precision of cellularization in *S. arctica,* focusing on the role of the MT network. Combining chemical and mechanical perturbations with imaging in *S. arctica*, we systematically investigate how MTs and membrane trafficking contribute to nuclear distribution at the cortex, where furrows are placed, how fast they progress, and finally the quality of daughter cells produced (Fig 1B and C). Analyzing how these distinct modules of cellularization come together in a concerted manner, we provide a better picture of *S. arctica* cellularization.

## Results and Discussion

### MTs are important for PM furrow spacing during cellularization

**Figure S1.**
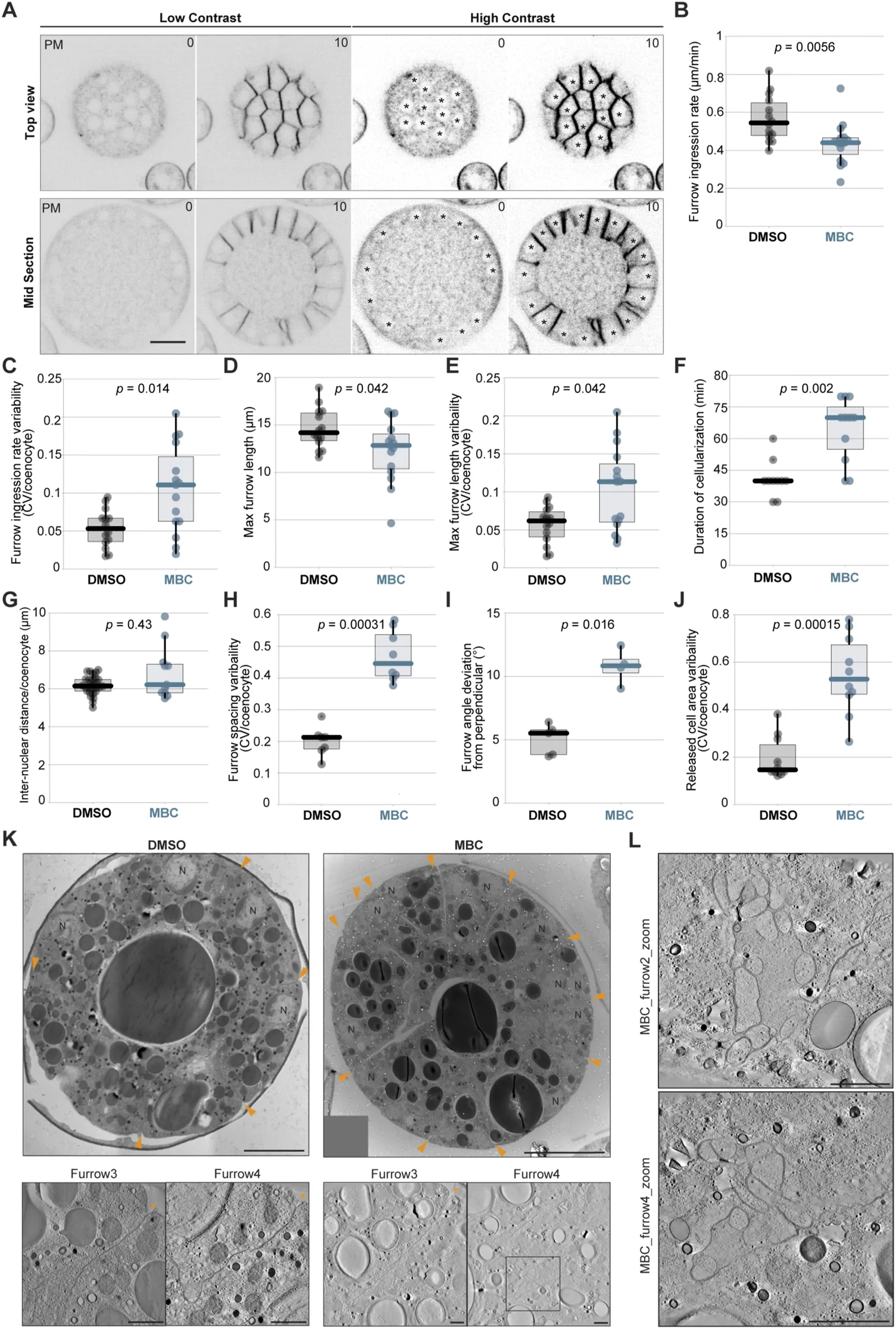
Cellularization dynamics and furrow organization in control and MT-depolymerized coenocytes. **(A)** Cellularizing control *S. arctica* coenocytes labeled with FM4-64, shown as a top view and an equatorial midsection at the onset of invagination (0 min) and 10 min later, each at low and high contrast display. Nuclei are visible as dye-excluded regions (asterisks). Scale bar, 10 μm. **(B)** Furrow ingression rate per coenocyte, taken as the slope of a linear fit through each furrow’s time points up to its maximum length. DMSO, 0.545 μm/min (n = 14); MBC, 0.441 μm/min (n = 15), 102 furrows from 29 coenocytes across three independent experiments. *P* = 0.0056. **(C)** Variability of furrow ingression rate within a coenocyte, from the rates in (B). DMSO, 0.053; MBC, 0.111, *P* = 0.014. **(D)** Maximum furrow length reached per coenocyte during tracking. Furrows tracked at fewer than three time points are excluded. DMSO, 14.19 μm (n = 14); MBC, 12.85 μm (n = 15), *P* = 0.042. Coenocytes were still elongating when tracking ended, so this is the length reached, not a final length. **(E)** Variability of maximum furrow length within a coenocyte, from (D). DMSO, 0.062; MBC, 0.113, *P* = 0.042. **(F)** Duration of cellularization, from the onset of membrane invagination to Flip. DMSO, 40 min (range 30–60, n = 10), MBC, 70 min (range 40–80, n = 11), *P* = 0.002. Flip occurred in all 21 coenocytes imaged. **(G)** Internuclear distance per coenocyte. DMSO, 6.15 μm (n = 28), MBC, 6.22 μm (n = 11), *P* = 0.43. Its variability is quantified in Fig 1F. **(H)** Variability of furrow spacing within a coenocyte. DMSO, 0.213; MBC, 0.446, *P* = 0.00031. The two distributions do not overlap. **(I)** Deviation of the invagination axis from perpendicular to the cortex, |angle - 90|, averaged per coenocyte. DMSO, 5.51 degrees (n = 5); MBC, 10.83 degrees (n = 4), 10 furrows per coenocyte; *P* = 0.016. Ingression depth did not differ between conditions. **(J)** Variability of released cell area within a coenocyte, segmented from live FM4-64 after cellularization. DMSO, 0.154 (n = 11); MBC, 0.520 (n = 12), 230 cells across conditions and three independent experiments. *P* = 0.00015. **(B, C, D, E, F, G, H, I, J)** Boxes show the median and interquartile range, and whiskers, 1.5 × IQR. Each point is one coenocyte unless stated otherwise. Mann–Whitney U test; exact *P*-values are given. **(K)** Top, TEM images of control and MBC-treated cells. Yellow arrowheads indicate invaginating furrows. N marks the nuclei. Scale bar, 5 μm. Bottom, single slices from TEM tomography images of invaginating furrows from control and MBC-treated cells. Scale bar, 1 μm. **(L)** Zoom-in views of convoluted membrane organization at furrow tips in MBC-treated furrows 2 (Fig 1J) and 4 (Fig S1K). Scale bar, 1 μm. Source data are available for this figure.

To test the role of MTs in cellularization, we focused on the onset of the postmitotic cellularization phase. We treated cellularizing coenocytes (34 h post-inoculation [hpi]) with the MT-depolymerizing agent carbendazim (MBC) for 2 h. As previously shown, unlike actin inhibition, MT depolymerization does not completely block cellularization in *S. arctica*, allowing us to monitor the altered dynamics in the presence of MBC (7). We used live-cell imaging with the lipophilic dye FM4-64 to monitor PM furrows and follow their ingression kinetics over time (Figs 1D and S1A). MT loss disrupted furrow ingression dynamics, producing uncoordinated ingression—in contrast to the consistent furrows observed in controls (Fig 1D and Video 1). Furrow ingression rates were reduced, progressing at 0.441 μm/min in MBC-treated cells versus 0.545 μm/min in controls (Figs 1E and S1B), with the variability of ingression rate within a coenocyte doubling upon treatment (Fig S1C). Notably, these invagination rates in *S. arctica* lie between those reported during *Drosophila* embryo cellularization, where slow and fast phase invaginations proceed at 0.2 ± 0.02 μm/min and 1.2 ± 0.10 μm/min, respectively, to achieve a final furrow length of ∼35 μm (29). MBC-treated coenocytes also failed to extend PM furrows to the same length as controls during tracking, achieving only 12.26 ± 3.24 μm compared with 14.62 ± 2.14 μm (Fig S1D), with similarly increased variability (Fig S1E). Overall, cellularization took longer, the interval from the onset of invagination to Flip rising from 40 to 70 min, delaying cell release (Fig S1F) (11).

Video 1 Time-lapse movie showing cellularization in DMSO (left) and MBC-treated (right) coenocytes labeled with FM4-64 and imaged at 10-min intervals. Scale bar, 10 μm. Download video

Beyond furrow ingression dynamics, MT depolymerization severely disrupted nuclear and furrow positioning. Nuclei were visualized as FM4-64–excluded regions in low- and high-contrast images (Fig S1A). Although mean internuclear spacing was not significantly different between conditions (6.15 versus 6.22 μm, Fig S1G), MBC-treated coenocytes exhibited dramatically increased variability in nuclear positioning (0.143 versus 0.268, Fig 1F), with nuclei ranging from properly positioned to severely mislocalized. In addition, MBC treatment disrupted the regular spacing of PM furrows observed in controls (Fig 1G and H). In control coenocytes, furrows were evenly distributed, enclosing one nucleus per cellular compartment (11, 26). MBC-treated coenocytes displayed significantly decreased (9.20–6.31 μm, Fig 1H) and irregular furrow spacing (0.213–0.446, Fig S1H), with furrows deviating further from the perpendicular to the cortex (5.51–10.83°, Fig S1I). This irregular cellularization was also reflected in the variable cell sizes produced after cellularization in MBC-treated coenocytes, in stark contrast to the uniform cell size in controls (0.154–0.520, Fig S1J and Video 1). To assess the ultrastructural basis of these defects, we obtained volumetric data of PM furrow organization. We performed focused ion beam scanning electron microscopy (FIB-SEM) and transmission electron microscopy tomography (TEM tomography). FIB-SEM of a cellularizing coenocyte enabled 3D segmentation and volumetric reconstruction of both the PM and nucleus of a newly forming cellular compartment (Fig 1I and Video 2). TEM tomography at higher resolution showed furrows with convoluted morphology in MBC-treated coenocytes, in contrast to the smooth, organized structure of control furrows (Figs 1J and S1K and L). Together, the altered ingression dynamics and the irregular nuclear and furrow positioning on disruption of MTs indicate that MTs sustain the fidelity of cellularization in *S. arctica*.

Video 2 FIB-SEM volume of cellularizing cell. Download video

### MTs track PM invaginations during cellularization

**Figure 2.**
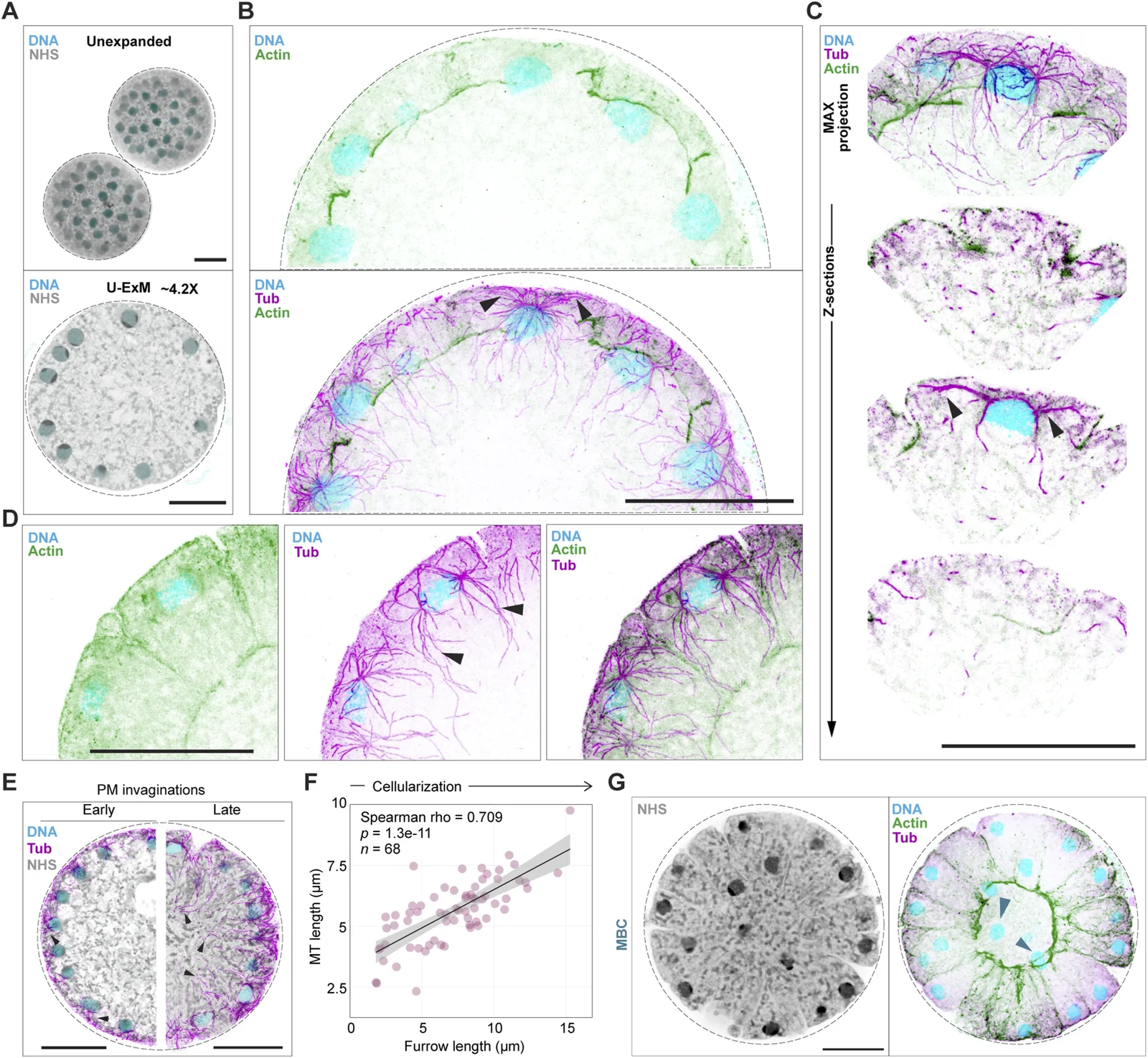
MT and actin cytoskeleton are organized along PM invaginations. **(A)** Maximum-intensity projections of nonexpanded and expanded *S. arctica* coenocytes labeled for DNA (blue) and NHS-ester (gray). **(B)** Top—maximum-intensity projection of an expanded coenocyte labeled for actin (green) and DNA (blue) during cellularization prefurrow ingression. Bottom—same image with all channels merged with tubulin (magenta). Arrowheads mark cortical (in B) or furrow-tracking MTs (in C, D). **(C)** Single cellularizing compartment, shown as a maximum-intensity projection (top) and as consecutive z-sections through the same region. **(D)** Maximum-intensity projection of a region of a cellularizing coenocyte at later stage of furrow ingression. The same region separated into channels: actin (green), tubulin (magenta), and merge. DNA is shown as a reference for nuclei. **(E)** MT organization at early and late stages of cellularization showcasing the extension of MT length during cellularization. DNA (blue), NHS-ester (gray), and tubulin (magenta). Arrowheads indicate MTs. **(F)** MT length in relation to PM furrow length. Spearman’s rho = 0.709, *P* = 1.3e−11, n = 68 coenocytes. **(G)** Maximum-intensity projections of expanded MBC-treated coenocytes labeled for actin (green), tubulin (magenta), and DNA (blue). Arrowheads indicate displaced nuclei. Scale bar, 10 μm. Scale bars are adjusted for expansion factor. Source data are available for this figure.

**Figure S2.**
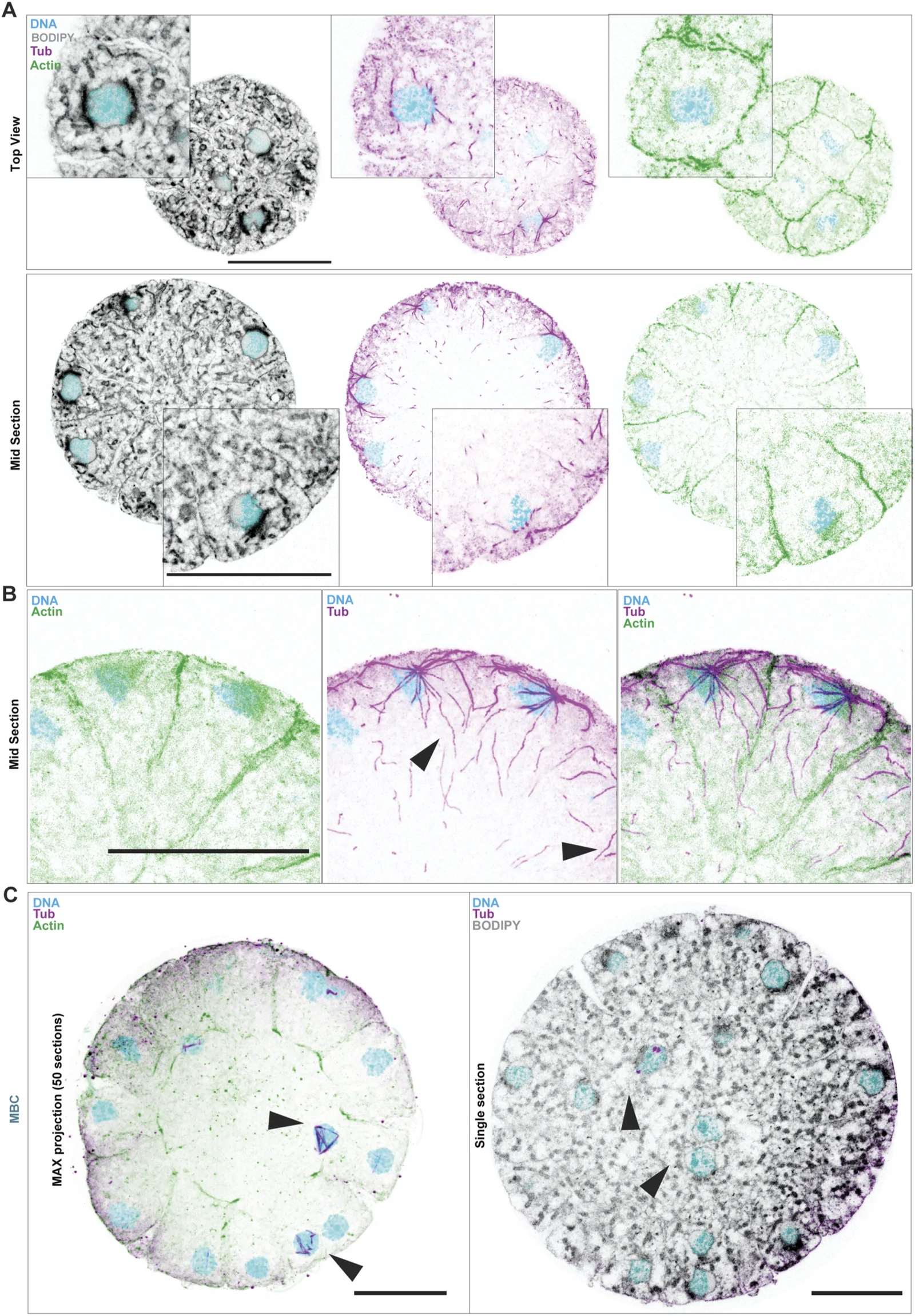
U-ExM of cellularizing and MT-depolymerized coenocytes. **(A)** Single-slice top view and equatorial midsection of an expanded cellularizing coenocyte, with zoom-ins on one forming compartment. BODIPY-ceramide (gray), tubulin (magenta), actin (green), and DNA (blue). **(B)** Second example of a late cellularizing coenocyte, shown as single channels and merge. **(C)** Two MBC-treated coenocytes: a maximum-intensity projection of 50 consecutive z-sections (left) and a single z-section of a second coenocyte (right). Tubulin signal persists around a subset of nuclei after MT depolymerization, and individual compartments enclose more than one nucleus. This observation is made on the single section and on the full volume in Video 5, not on the projection, because nuclei at different depths overlap in projection. Scale bar, 10 μm. Scale bars are adjusted for expansion factor.

To determine how MTs contribute to furrow positioning and organization, we examined their spatial relationship with the PM during cellularization using ultrastructure expansion microscopy (U-ExM) on cellularizing *S. arctica* coenocytes (Fig 2A). U-ExM enables efficient immunolabeling in cell-walled microbial eukaryotes by permeabilizing their protective cell layer during the expansion process, thereby improving antibody accessibility (28, 30, 31). Leveraging this approach, we applied the recently developed U-ExM–compatible HAK-actin probe together with anti-tubulin antibodies to simultaneously visualize both cytoskeletal networks throughout cellularization (Figs 2B–D and S2A; Video 3 and Video 4) (32). This represents a significant advance over standard immunofluorescence protocols, which fail to label or resolve cytoskeletal structures in *S. arctica*. As a reference for overall cellular organization, we combined these with membrane (BODIPY-ceramide), DNA, and protein pan-labeling (NHS-ester) (Figs 2 and S2).

Video 3 U-ExM volume of cellularizing compartments. tubulin (magenta), DNA (blue), and NHS-ester pan-labeling (gray). Download video

Video 4 U-ExM volume of an untreated coenocyte at an early stage of PM invagination (cropped), stepped section by section. Both panels show the same crop and the same sections in two channel combinations: left, NHS-ester (gray) and tubulin (magenta); right, DNA (blue), tubulin (magenta), and actin (green). Scale bar, 10 μm, adjusted for expansion factor. Download video

Actin localized at the base of membrane furrows, marking the boundaries of the cellularizing compartments throughout the invagination process (Fig 2C and D). Although distinct contractile rings were not resolved, actin consistently accumulated at the invagination front. Notably, actin was also visible at the basal opening of nascent compartments that remained continuous with the shared cytoplasm (Fig 2C and D). In previous studies, actin accumulation has been interpreted as a marker of completed membrane sealing (11). However, our observations indicate that actin enrichment precedes final compartment closure, suggesting that membrane sealing occurs later than previously assumed. Consequently, the duration of the transient multicellular state in *S. arctica* might have been overestimated in earlier interpretations.

At early stages, MTs extend from nucleus-associated MTOCs radially along nuclei toward the center of the coenocyte and toward the cell cortex (Fig 2B and C) (28). As furrow formation progressed, we observed that MTs oriented toward the cell cortex and furrow-tracking MTs aligned along actin-marked PM invaginations (Fig 2D, arrowheads; a second example of late cellularization is shown in Fig S2B). Quantitative analysis of MT networks showed that MT length scaled with furrow depth (Spearman’s rho = 0.709, *P* = 1.3 × 10^−11^, n = 68 coenocytes), consistent with MTs elongating in coordination with PM invagination (Fig 2E and F). Although this correlation suggests a role for MTs in furrow progression, it remains unclear whether this involves active polymerization at the furrow front or the use of preformed MT tracks. To further assess the requirement for MT dynamics in furrow organization, we analyzed MBC-treated coenocytes by U-ExM. Actin localization at the furrow base persisted, indicating that furrow formation can, as previously shown, proceed independently of MTs (Fig 2G). However, furrow positioning was severely affected, as shown in Fig 1. Furrows were often misaligned, and individual compartments were observed to enclose more than one nucleus (Fig S2C and Video 5). Nuclei were also seen trailing between furrows or lying beneath partially formed compartments (Video 5).

Video 5 U-ExM volume of a whole MBC-treated coenocyte, stepped through the complete z-series. BODIPY (gray), tubulin (magenta), and DNA (blue). Nuclei are unevenly distributed, a subset retains a short tubulin-positive remnant despite MT depolymerization, and individual compartments enclose more than one nucleus. Scale bar, 10 μm, adjusted for expansion factor. Download video

These defects indicate that although furrow initiation is actin-driven, MTs are required to sustain nuclear and furrow positioning. In their absence, cellularization proceeds but generates aberrant compartments with irregular geometry and compromised nuclear allocation. Taken together, these observations show that MTs in *S. arctica* reorganize during cellularization, transitioning from radial arrays around nuclei to furrow-aligned bundles that extend with membrane invagination. This dynamic reorganization, combined with actin accumulation at the furrow base, points to a coordinated cytoskeletal architecture. This coordination between actomyosin-dependent furrows and MT networks likely reflects a conserved cytoskeletal role in organizing cellular domains within a common cytoplasm, parallel to mechanisms observed in early-stage insect embryos.

### Nucleus–MTOC positioning guides furrow placement

**Figure 3.**
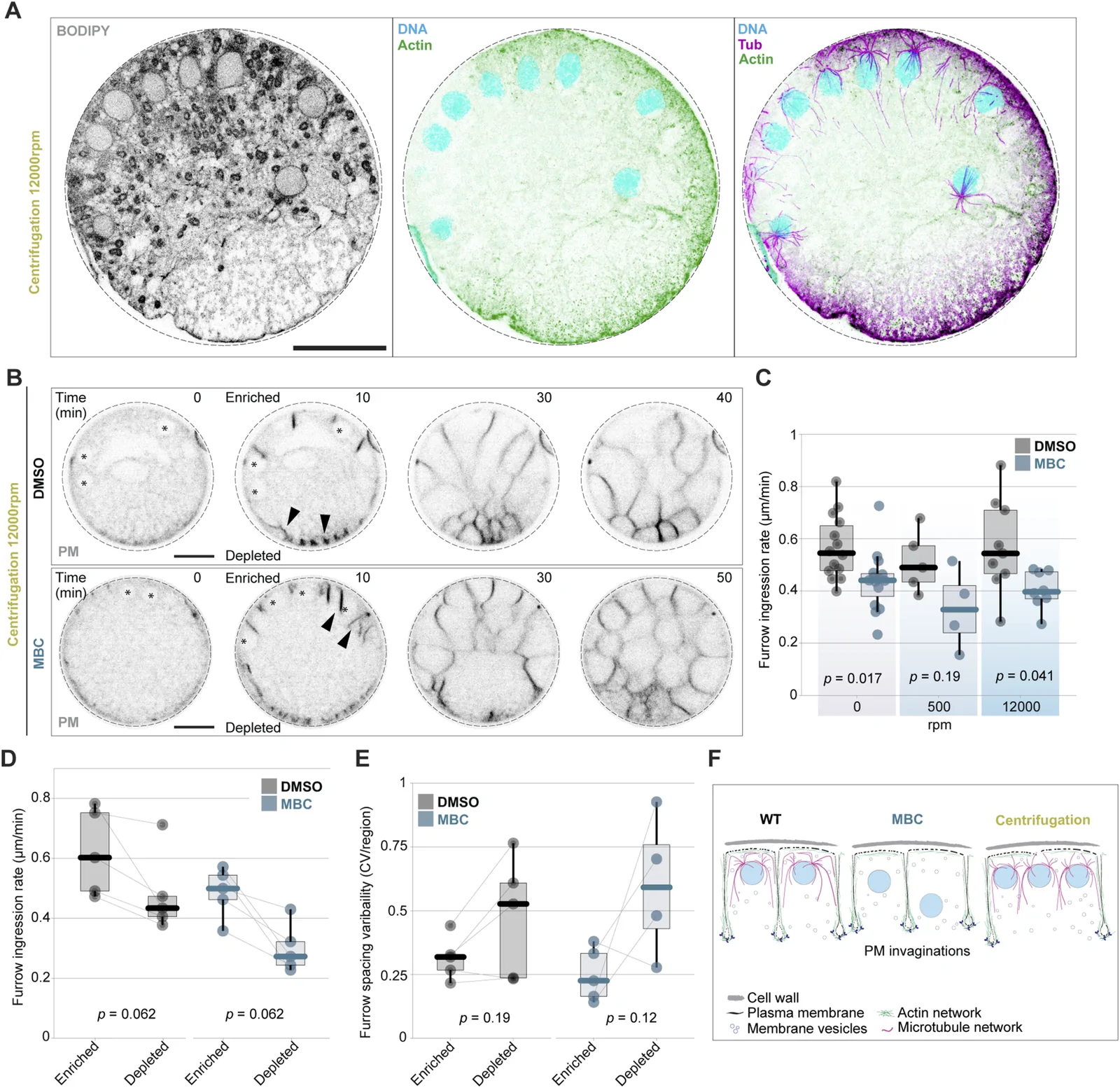
Nuclear positioning ensures fidelity of cellularization. **(A)** Maximum-intensity projections of U-ExM images of coenocytes post-centrifugation, labeled for membrane (gray), actin (green), tubulin (magenta), and DNA (blue). Scale bar, 10 μm, adjusted for expansion factor. **(B)** Time-lapse images of FM4-64–labeled coenocytes showing PM furrow ingression at 10-min intervals in centrifuged coenocytes treated with DMSO or MBC. Scale bar, 10 μm; asterisks indicate nuclei. **(C)** Furrow ingression rate per coenocyte across spin conditions, DMSO and MBC at 0, 500, and 12,000 rpm; 627 length measurements from four independent experiments. DMSO versus MBC within each spin condition, three planned comparisons with the Benjamini–Hochberg correction: 0 rpm, *P* = 0.017; 500 rpm, *P* = 0.19; 12,000 rpm, *P* = 0.041. **(D)** Furrow ingression rate by region, 12,000 rpm; 40 furrows. DMSO, enriched 0.600 μm/min and depleted 0.448 μm/min; MBC, enriched 0.493 μm/min and depleted 0.279 μm/min; paired Wilcoxon *P* = 0.062 in both, at the floor of the test. **(E)** Variability of furrow spacing within each region 10 min after the onset of invagination. DMSO, *P* = 0.19; MBC, *P* = 0.12. **(F)** Cartoon showing the impact of perturbations (MBC and centrifugation) on nuclear and cytoskeletal organization during PM invagination. Boxes show the median and interquartile range, and whiskers, 1.5 × IQR. Region comparisons are paired within a coenocyte, Wilcoxon signed-rank test; comparisons between drugs are performed by the Mann–Whitney U test. Exact *P*-values are given. Source data are available for this figure.

Although MBC treatment demonstrates that MT disruption leads to both nuclear and furrow misplacement, it remains unclear whether nuclei guide furrow positioning or newly forming furrows position the nuclei instead. To assess the role of nuclear positioning in furrow organization, we centrifuged cellularizing coenocytes using an Eppendorf 5425/5430 microcentrifuge with an FA-45-30-11 fixed-angle rotor, to induce asymmetric displacement of nuclei along the cortex (Fig 3A). This approach builds on previous work where we used centrifugation to perturb the spatial relationship between nuclei and the cortex and demonstrated that cellularization in *S. arctica* is sensitive to local nucleocytoplasmic ratios (26). Centrifugal displacement is partially penetrant, with ∼40% of centrifuged coenocytes showing irregular PM invaginations and about 10% undergoing lysis (26). Analyses were therefore restricted to coenocytes showing clear nuclear displacement.

**Figure S3.**
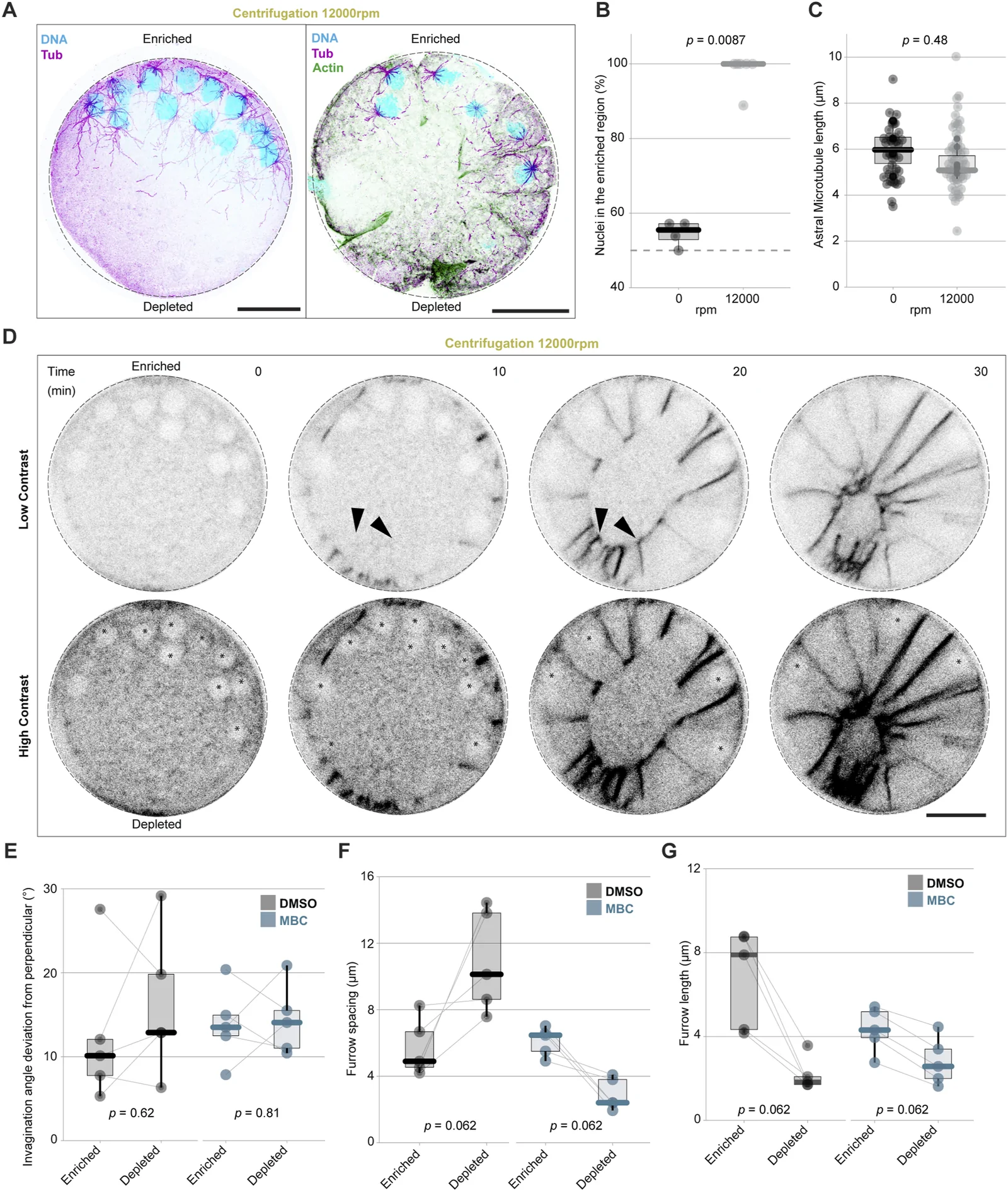
Centrifugation displaces nuclei and their MTs without destroying them. **(A)** Maximum-intensity projections of U-ExM images of centrifuged coenocytes with the nuclear-enriched and nuclear-depleted regions indicated. DNA (blue), tubulin (magenta), actin (green). Scale bar, 10 μm, adjusted for expansion factor. **(B)** Percentage of nuclei in the enriched region per coenocyte, before and after centrifugation. Uncentrifuged, 55% (n = 4); 12,000 rpm, 100% (n = 6), *P* = 0.0087. The dashed line marks an even distribution. **(C)** Length of the longest MT of each nuclear aster, measured on maximum-intensity projections of 15 consecutive z-sections and corrected for the expansion factor. Small pale points are individual MTs, and large solid points are the per-coenocyte median, which is the replicate unit and the only value used for the test; 81 MTs from 10 coenocytes. Uncentrifuged, 5.97 μm (n = 4); centrifuged, 5.08 μm (n = 6), *P* = 0.48. Every nucleus retained an aster. In the nuclear-depleted cortex of the same coenocytes, tubulin was present only as short fragments, a median of three per coenocyte and 0.95 μm long (see SourceData for Fig S3), which are different objects from an aster and are not tested against it. **(D)** Live FM4-64 imaging of a centrifuged coenocyte from the onset of invagination to 30 min, shown at low and high display contrast. Nuclei are visible as dye-excluded regions (asterisks). Scale bar, 10 μm. **(E)** Deviation of the invagination axis from perpendicular to the cortex by region; 145 furrows. DMSO, *P* = 0.62; MBC, *P* = 0.81. Boxes show the median and interquartile range, and whiskers, 1.5 × IQR. Region comparisons are paired within a coenocyte, Wilcoxon signed-rank test; comparisons between drugs are by the Mann–Whitney U test. Exact *P*-values are given. **(F)** Furrow spacing in the nuclear-enriched and nuclear-depleted region of the same coenocyte 10 min after the onset of invagination, 12,000 rpm. Each point is one coenocyte, and lines join the two regions of that coenocyte; 135 gaps from 10 coenocytes. DMSO, enriched 4.90 μm and depleted 10.13 μm; MBC, enriched 6.47 μm and depleted 2.41 μm; paired Wilcoxon *P* = 0.062 in both, which is the smallest value the test can return at n = 5. Spacing is measured between furrows that progressed far enough to be traced, and the nuclear-depleted region carries additional small indentations that do not sustain ingression. **(G)** Furrow length by region. DMSO, enriched 7.50 μm and depleted 2.14 μm; MBC, enriched 3.83 μm and depleted 2.95 μm; paired Wilcoxon *P* = 0.062 in both, at the floor of the test. Source data are available for this figure.

In our experiments, centrifugation resulted in nuclei accumulating on one side of the coenocyte, creating a strong spatial asymmetry in nuclear distribution at the cortex (Figs 3A and S3A), with one hemisphere holding all of the nuclei after centrifugation as compared to 55% before (Fig S3B). U-ExM imaging, including BODIPY-ceramide labeling for enhanced membrane visualization, confirmed this asymmetric nuclear redistribution (Fig 3A). MTs were relocated with the nuclei as every nucleus retained the associated MTOC and MT network, and the length of the longest MT of each network was unchanged by centrifugation (5.97 against 5.08 μm, Fig S3C). Throughout these experiments, nuclei are resolved in single optical sections as regions from which FM4-64 is excluded and are visible as such at both low and high display contrast (Fig S3D). In the nuclear-depleted cortex of the same coenocytes, tubulin was present only as some short fragments, a median of three per coenocyte with a median length of 0.95 μm (SourceData for Fig S3). In nuclear-enriched regions, furrows were misaligned, frequently enclosing multiple nuclei per compartment rather than the single nucleus observed in controls (Fig 3B and Video 6).

Video 6 Time-lapse movie showing cellularization in control (500 rpm, left) and post-centrifugation (12,000 rpm, right) in MBC-treated coenocytes labeled with FM4-64 and imaged at 10-min intervals. Scale bar, 10 μm. Download video

After centrifugation, overall furrow ingression proceeded with kinetics comparable to controls, confirming that the core machinery for membrane trafficking and actin-driven invagination remained functional (Figs 3B and S3D), with ingression rate unaffected by centrifugation in both DMSO (0 versus 12,000 rpm, *P* = 0.975)- and MBC (0 versus 12,000 rpm, *P* = 0.681) (Fig 3C)-treated coenocytes. Comparing local dynamics, although the angle of invagination did not differ between the two regions (Fig S3E) the ingression rate was lower where nuclei were absent (0.448 against 0.600 μm/min, Fig 3D). This local slowing of ingression in the nuclear-depleted regions in control and MBC-treated cells may be due to a combination of reduced MTs and displacement of membrane pool (Fig 3A and D).

Furrow spatial patterning was dramatically altered: in nuclear-depleted cortical regions, furrow initiations were more frequent and closely clustered but failed to progress deeply (Figs 3B and S3D). Among the furrows that did progress, spacing was more variable in the nuclear-free region than in the nuclear-enriched half of the same coenocyte (Fig 3E). Because furrow spacing is reliably quantifiable only for furrows that progress, the effective furrow spacing in this nuclear-depleted region appeared wider and was abolished upon MBC treatment (Fig S3F). Taking into account the high number of furrow initials in the nuclear-depleted region (Figs 3B and S3D), and furrow separation in the region enriched with nuclei suggests a minimum furrow exclusion zone around individual nuclei and their MT networks. Furrows in nuclear-depleted regions reached shorter lengths (2.14 against 7.50 μm, Fig S3G), forming asymmetrically longer compartments on the nuclear-enriched side. This difference in furrow length was lowered upon MBC treatment confirming the role of nucleus-associated MT networks in maintaining the synchronous invagination and uniform cellular partitioning. With centrifugation, though the nucleus-associated MT networks also migrate, the regular spacing of the cytoskeletal network at the cortex was disrupted, and furrows no longer exhibited the uniform spacing characteristic of control coenocytes, suggesting indeed that MT-defined nuclear territories serve as spatial landmarks that define the coordinates of membrane invagination. The mechanism underlying this spatial control remains to be fully resolved: it may involve MT-mediated physical coupling between nuclei and cortical membrane domains, localized signals emanating from nucleus-associated MTOCs, local nucleocytoplasmic ratios, or a combination of these factors. Taken together, results from the centrifugation and MBC perturbations support a model in which the nucleus–MTOC unit functions as an organizational hub: (i) MTs maintain nuclear distribution to establish properly spaced cortical domains where (ii) actin-driven membrane invagination proceeds with correct geometry and nuclear allocation (Fig 3F).

### *S. arctica* cellularization relies on membrane trafficking

**Figure S4.**
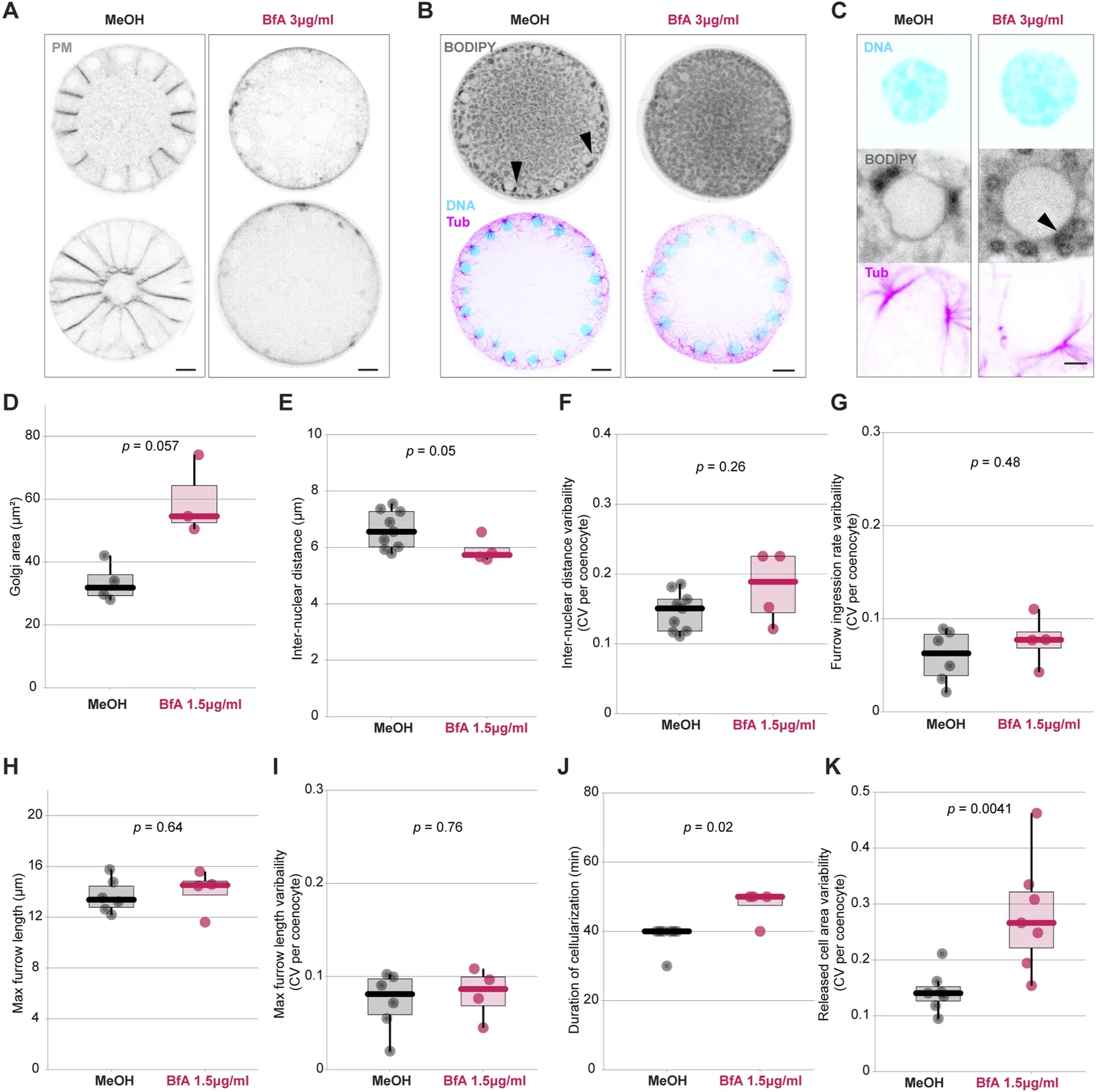
Dose-dependent effects of BfA on cellularization. Panels (A, B, C) are images of cells treated with 3 μg/ml BfA; the quantification in (D) is at 1.5 μg/ml BfA, the only dose at which Golgi area was measured, and no measurement is reported at 3 μg/ml. **(A)** Live FM4-64 imaging of coenocytes treated with methanol or 3 μg/ml BfA; invagination does not proceed. Scale bar, 10 μm. **(B)** U-ExM of methanol- and 3 μg/ml BfA-treated coenocytes. BODIPY (gray), DNA (blue), and tubulin (magenta). Scale bar, 10 μm, adjusted for expansion factor. **(C)** Zoom-ins on the Golgi from the images in (B). **(D)** Golgi area per coenocyte, methanol versus 1.5 μg/ml BfA; 21 Golgi from seven coenocytes. Methanol, 32.97 μm2 (n = 4); BfA, 54.43 μm2 (n = 3), *P* = 0.057, which is the smallest value a Mann–Whitney test can return at this n and denotes complete separation of the two groups. Normalized intensity was measured on the same images and does not separate (*P* = 0.11); it is not shown. **(E)** Internuclear distance per coenocyte; 126 distances. Methanol, 6.56 μm (n = 9); BfA, 5.73 μm (n = 4), *P* = 0.050. **(F)** Variability of internuclear distance within a coenocyte. Methanol, 0.151; BfA, 0.189, *P* = 0.26. Contrast with Fig 1F, where MT depolymerization nearly doubles this variability (*P* = 5.6e-06). **(G)** Variability of furrow ingression rate within a coenocyte. Methanol, 0.063; BfA, 0.077, *P* = 0.48. **(H)** Maximum furrow length reached per coenocyte; 55 furrows. Methanol, 12.64 μm (n = 9); BfA, 14.47 μm (n = 5), *P* = 0.64. **(I)** Variability of maximum furrow length within a coenocyte. Methanol, 0.081; BfA, 0.086, *P* = 0.76. **(J)** Duration of cellularization, from the onset of membrane invagination to Flip. Methanol, 40 min (n = 7); BfA, 50 min (n = 4), *P* = 0.020. Flip was observed in every coenocyte scored. **(K)** Variability of released cell area within a coenocyte; 196 cells. Methanol, 0.140; BfA 0.266, *P* = 0.0041 (n = 7). Boxes show the median and interquartile range, and whiskers, 1.5 × IQR. Mann–Whitney U test; exact *P*-values are given. Source data are available for this figure.

**Figure 4.**
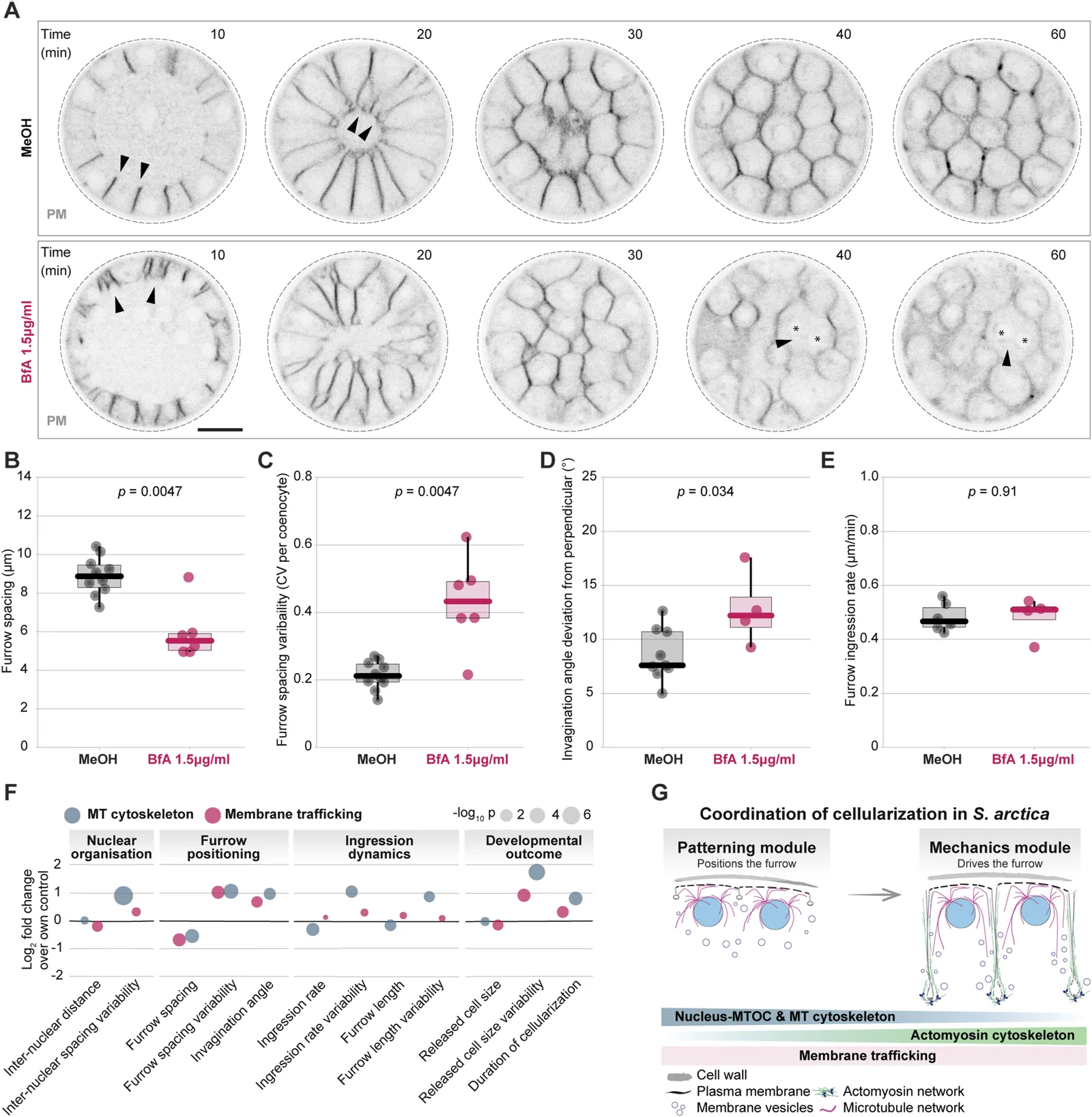
BfA inhibition leads to furrow mispositioning. **(A)** Membrane invagination dynamics in control (methanol) and BfA (1.5 μg/ml)-treated coenocytes labeled with the FM4-64 membrane dye. Scale bar, 10 μm. Asterisks mark two nuclei in one compartment in BfA-treated cells. **(B)** Furrow spacing per coenocyte, measured as in Fig 1H; methanol, 8.87 μm (n = 10); BfA, 5.53 μm (n = 6), 251 gaps from 16 coenocytes. *P* = 0.0047. **(C)** Variability of furrow spacing within a coenocyte, from the same measurements. Methanol, 0.212; BfA, 0.433, *P* = 0.0047. Measured the same way in both experiments, the two perturbations are indistinguishable from one another (MBC, 0.446 versus BfA, 0.433, *P* = 0.66) and so are the two controls (DMSO, 0.213 versus methanol, 0.212, *P* = 0.67). **(D)** Deviation of the invagination axis from perpendicular to the cortex, averaged per coenocyte; methanol, 7.60 degrees (n = 9); BfA, 12.21 degrees (n = 4), 127 furrows from 14 coenocytes. *P* = 0.034. **(E)** Furrow ingression rate per coenocyte, taken as in Fig S1B; 55 furrows. Methanol, 0.467 μm/min (n = 6); BfA, 0.510 μm/min (n = 4), *P* = 0.91. **(F)** 10 measurements of cellularization compared between the two perturbations, each as log2 fold change against its own control (MBC over DMSO, BfA over methanol). Rows are grouped by phenotype. Color indicates the machinery, and point size indicates –log10 p. **(G)** Model summarizing how the nucleus–MTOC axis and the MT cytoskeleton position furrows, and how actomyosin and membrane trafficking drive their ingression, separated into a patterning module and a mechanics module. Boxes show the median and interquartile range, and whiskers, 1.5 × IQR. Mann–Whitney U test; exact *P*-values are given. Source data are available for this figure.

Although these experiments define how cellularization is spatially patterned, the cellular machinery driving membrane invagination itself remained to be identified. The previous experiments show that furrow invagination proceeds even when furrows are mispositioned. This indicates that the processes of new membrane addition and furrow positioning are controlled independently. To identify the cellular processes driving furrow invagination, we examined the role of membrane trafficking. In early *Drosophila* embryos, the membrane expands from a reservoir of microvilli localized at the apical cortex (33, 34). The first phase of this membrane invagination is supplemented partly by Golgi-derived vesicles. These vesicles are transported in a MT-dependent manner and are stalled with Colcemid or colchicine treatment, which eventually prevented furrow invagination (18, 23, 35). It is unclear whether such a membrane reservoir is present at the *S. arctica* cortex where the furrows are first initiated. Brefeldin A (BfA) treatment in *Drosophila* embryos inhibits furrow progression in the final stage of cellularization (15, 18). To test whether membrane trafficking contributes to furrow dynamics in *S. arctica*, we treated coenocytes with BfA, which inhibits protein transport from the endoplasmic reticulum to the Golgi apparatus. High-dose (3 µg/ml) BfA treatment interferes with and in some cells even blocks cellularization, confirming that Golgi-mediated vesicle trafficking is required for cellularization (Fig S4A–C). To examine the specific effects on furrow organization, we used live imaging to follow the dynamics of cellularization in *S. arctica* coenocytes upon mild membrane trafficking impairment in the presence of 1.5 μg/ml BfA (Fig 4A).

Using this milder BfA treatment, we find that membrane furrows are still formed and can ingress at WT rates; however, they appear mispositioned like those seen upon MT depolymerization (Fig 4A). At this dose, the Golgi was enlarged (32.97–54.43 μm^2^, Fig S4D). Furrow spacing was reduced (8.87–5.53 μm, Fig 4B) and became markedly less regular (0.212–0.433, Fig 4C), and furrows deviated further from perpendicular to the cortex (7.60–12.21°, Fig 4D). Nuclear positioning, in contrast, was not detectably affected as neither internuclear distance nor its variability differed from controls (Fig S4E and F). Ingression rate (Fig 4E), its variability (Fig S4G), and the maximum length reached by furrows (Fig S4H and I) were likewise comparable to controls. Cellularization nonetheless took longer to complete (from 40 to 50 min, Fig S4J), and the cells released at the end were of more variable size (0.140–0.266, Fig S4K).

In summary, our results indicate that nucleus-associated MTOCs serve as potential signaling hubs to define the coordinate system for nuclear territories within a shared cytoplasm. Previously, we showed that like in *Drosophila* and *S. punctatus,* a threshold local nuclear density at the cortex triggers cellularization in *S. arctica* (3, 4, 9, 26). How this local density of nuclei in a coenocyte is counted and how each nuclear territory is set remain unknown. Are there interactions between MTs or MTOCs from neighboring nuclei? Are these interactions dependent on the cell cycle stage as nuclei cycle between one or two MTOCs? MTOCs through their close association with nuclei, defined number per nucleus, and ability to organize and remodel MT networks in response to cellular and cortical cues make them prime candidates for signaling spatial information in *S. arctica*. In *D. melanogaster*, for instance, nuclei are positioned by antiparallel crosslinking of astral MTs by kinesin and PRC1 (22, 36). In chytrid *S. punctatus*, nuclear-associated centrosomes interact with the cortex, initiating furrow formation. In *A. thaliana*, astral MTs guide nuclear spacing and cellularization seeds (8). Here, we showed that MT networks set nuclear positioning, which in turn specified or at least reinforced furrow positioning, and ensured coordinated furrow ingression. Distinct from chytrids where nuclei are carried by ingressing furrows, furrows are placed in between nuclei. Actomyosin activity and vesicle delivery are the two forces that drive furrow ingression and eventual closure, and unlike fly embryos which stall with Colcemid treatment, in *S. arctica* they are capable of operating in the complete absence of MTs (17, 19, 20). Depolymerizing actin, or inhibiting Arp2/3, formins, or myosin II, each abolished PM invagination (11). Actin delineated furrow boundaries, and accumulated at the invaginating front even in the absence of MTs. However, the perfect coordination of furrows in space and time to define autonomous nuclear territories was lost. A distinct outcome was seen with the loss of membrane delivery, where a high BfA dose blocked the invagination process. In contrast, with a milder dose, cellularization proceeded inconsistently, producing variable daughter cells, suggesting that membrane delivery cooperates with the cytoskeletal machinery to support specification and furrow invagination (Fig 4F and G). How the disruption of membrane trafficking affects coordination and whether this impacts the nucleus–MTOC signaling, through their interaction with the Golgi, early on or is a much downstream effect are open for future investigation. Overall, cellularization in *S. arctica* separates into a patterning module, in which the nucleus–MTOC axis defines the fidelity of cell specification, and a mechanics module, in which the actomyosin and membrane delivery machinery provide the driving forces for a coordinated ingression at a whole coenocyte scale (Fig 4G). On this basis, the cellularization process seems largely conserved across opisthokonts (3, 4, 9, 19). Comparison of the minor variations between Ichthyosporea, chytrid, and animal models will inform further on the furrow specification process. Whether this separation of cytoskeletal roles extends to other eukaryotic lineages, such as amoebozoa, plants, and apicomplexans, many of which show multinucleate life cycle stages and the cellularization process, remains to be seen (1, 2). Nonetheless, it is clear that future analysis into cellularization mechanisms must take a more holistic approach addressing how the different molecular and cytoskeletal toolkits function together rather than investigating them in isolation. Taken together, our ongoing work on cytoskeletal organization directing life cycle stages in *S. arctica* and the findings from this study lay the groundwork for in-depth future analysis of cellularization mechanisms in an emerging opisthokont model.

## Materials and Methods

### *S. arctica* culture and growth conditions

*S. arctica* was provided by the laboratory of Omaya Dudin. *S. arctica* cultures were maintained at 17°C in marine broth (MB; Difco, 37.4 g l^−1^) in culture flasks (Falcon 25-cm^2^ Rectangular Canted Neck Cell Culture Flask with Vented Cap, product number 353109; Corning) and synchronized as previously described (7, 26, 27). Briefly, for synchronization, 1/16 MB was prepared by diluting MB in artificial seawater (Instant Ocean, 37 g l^−1^). Cultures were diluted 1:50 in 1/16 MB and grown for 5 d. 5-d-old cultures were inoculated 1:60–1:70 in cold MB to get a synchronized culture. To obtain coenocytes undergoing cellularization, coenocytes were fixed within 35.5–38 hpi. For experiments, *S. arctica* cultures were diluted 1:200 every week, whereas for maintenance, coenocytes were diluted 1:1,000 every 2 wk and restarted from a cryopreserved stock in 10% DMSO every 6 mo.

### Effect of inhibitor treatments on *S. arctica* cellularization process

For MT perturbation during cellularization, coenocytes grown for 34 h were treated with 25 μg ml^−1^ of MBC (378674; Sigma-Aldrich, using a 1:100 dilution from a freshly prepared stock solution at 2.5 mg ml^−1^ in DMSO for 2 h). Cells were collected and fixed at 35.5, 36, 37, and 38 hpi. To inhibit membrane trafficking, coenocytes were treated with 1.5 or 3 μg ml^−1^ BfA (catalog # 00-4506; eBioscience Brefeldin A Solution 1,000× in methanol) at 34 hpi with methanol at 1:2,000 as the control. The 3-μg ml^−1^ dose interferes with and in some coenocytes blocks cellularization and is used qualitatively only; all BfA measurements reported in this study were made at 1.5 μg ml^−1^.

### Chemical fixation and staging for cellularization by phalloidin and Hoechst staining

Cell culture flasks were scraped, and the suspension was collected into 15-ml Falcon tubes and centrifuged using an Eppendorf 5425/5430 microcentrifuge with an FA-45-30-11 fixed-angle rotor, for 5 min at 500 rpm at 17°C. The supernatant was removed, and coenocytes were transferred to 1.5-ml microfuge tubes and fixative was added for 30 min. The cells were fixed with 4% formaldehyde, 0.025% glutaraldehyde (Grade I, 25% in water, G5882-50ML; Sigma-Aldrich) in 250 mM sorbitol solution. The fixed cells were washed twice with 1× phosphate-buffered saline (PBS) and resuspended in 100 μl of 1XPBS. For staining of actin and DNA, a staining solution containing Hoechst 33342 (20 mM, 1,000× stock) at 1:500 and Alexa Fluor 568 or 488 phalloidin (400× stock, A12380 or A12379; Invitrogen) at 1:200 dilution was prepared in 1x PBS. Briefly, 2 μl of this staining solution was placed on a glass slide and 2 μl of the fixed cell suspension was added and mixed into the staining solution, immediately covered with an 18 × 18 mm diameter coverslip, and sealed with nail polish. Glass slides were incubated for 5 min at room temperature, covered from light, and imaged immediately after.

### Ultrastructure expansion microscopy

U-ExM was performed as previously described (28, 32, 37). Briefly, the cells were fixed as above. The fixed cells were then allowed to attach to 6- or 12-mm poly-D-lysine–coated (P1024-10MG; Sigma-Aldrich) coverslips for 1 h. For crosslinking with the HAK-actin probe (JASP-HA, Spirochrome), attached coenocytes were washed once with a drop of 1x PBS and then permeabilized with 1% PBS-T (Tween-20) for 30 min by adding a drop onto each coverslip (32). After permeabilization, coverslips were incubated with the HAK-actin probe at 1:500 dilution in 1x PBS (15-20 μl per 6-mm coverslip) for 45 min at room temperature. Then, the HAK-actin incubation solution was removed and coverslips were washed with 1x PBS. To crosslink the HAK-actin probe, coenocytes were fixed again (4% formaldehyde + 0.025% glutaraldehyde + 250 mM sorbitol in PBS, freshly prepared) for 10 min at room temperature. The fixative solution was washed twice with 1x PBS. This was followed by anchoring in 1% acrylamide/0.7% formaldehyde solution overnight at 37°C. A monomer solution (19% [wt/wt] sodium acrylate [Chem Cruz, 7446-81-3; AKSci], 10% [wt/wt] acrylamide [A4058; Sigma-Aldrich], 0.1% [wt/wt] N, N′-methylenebisacrylamide [M1533; Sigma-Aldrich] in PBS) was used for gelation, and gels were allowed to polymerize for 1 h at 37°C in a moist chamber. For denaturation, gels were transferred to the denaturation buffer (50 mM Tris, pH 9.0, 200 mM NaCl, 200 mM SDS, pH adjusted to 9.0) for 15 min at room temperature and then shifted to 95°C for 1 h. After denaturation, expansion was performed with several washes in water. After expansion, gel diameter was measured and used to determine the expansion factor. For all U-ExM images, scale bars indicate actual size; rescaled for gel expansion factor.

For immunostaining, all antibodies were prepared in 3% BSA in 1x PBS with 0.1% Tween-20. Primary antibodies (alpha- and beta-tubulin antibodies, AA344 and AA345; ABCD Antibodies) were used at 1:500, and the anti-HA antibody (HA-Tag Monoclonal Antibody, 2-2.2.14, catalog # 26183; Invitrogen) was used at 1:250 and incubated overnight at 37°C. This was followed by three washes for 10 min at room temperature and the addition of the secondary antibody. Goat anti-mouse secondary antibody conjugated to Alexa Fluor 488 (A-11001; Thermo Fisher Scientific) or 647 (A-21235) was used at 1:250, and goat anti-guinea pig secondary antibody conjugated to Alexa Fluor 488 (A-11073; Thermo Fisher Scientific) or 647 (A-21450; Thermo Fisher Scientific) was used at 1:500. Pan-labeling of U-ExM was done at 1:500 with DyLight 405 (46400; Thermo Fisher Scientific) or Alexa Fluor NHS-Ester 594 (A20004; Thermo Fisher Scientific) in 1× PBS or NaHCO3 for 1.5–2 h or overnight (1:700 dilution). For membrane labeling, gels were stained with BODIPY TR ceramide (D7540; Thermo Fisher Scientific, 2 mM stock in DMSO) at 1:500–1:1,000 dilution in 1× PBS for 2 h at room temperature. DNA was stained with Hoechst 33352 at a final concentration of 0.4 μM.

### Light microscopy

Immunolabeled U-ExM gels were placed in poly-L-lysine–coated ibidi chamber slides (two-well, ibidi 80286; four-well, ibidi 80426). Gels were cut to fit the ibidi chambers and added onto the wells. The gels were overlaid with water to prevent drying or shrinkage during imaging. Gels were imaged using a Nikon CSU-W1 SoRa microscope with an Apo LWD 40x WI Lambda-S/1.15/0,60/water immersion objective with the lasers 405 nm (120 mW), 488 nm (200 mW), 561 nm (150 mW), and 638 nm (200 mW), or a Nikon A1 confocal microscope with a CFI Apo LWD Lambda-S 40x/1.15 WI 0,60 WD = 610 μm objective and LU-N4 405/488/561/640 laser unit. Z-stacks were acquired with 130 (half coenocyte)–300 (whole coenocyte) slices using a z-step size of 0.5 μm and a zoom factor of 2.

### Live-cell imaging

For live-cell imaging, cells were synchronized as described above and grown for 34 hpi in MB at 17°C in 4-well ibidi chamber slides (80426; ibidi). To induce MT depolymerization and monitor cellularization dynamics, coenocytes were treated for 2 h with either DMSO as a control or the MT inhibitor MBC (Sigma-Aldrich, 2.5 mg/ml stock in DMSO, final concentration 25 μl/ml). To perturb membrane trafficking during cellularization, coenocytes were treated for 2 h with either methanol at 1:2,000 dilution as a control or the membrane trafficking inhibitor BfA at 1.5 μg/ml or 3 μg/ml (1,000× stock in methanol). The membrane dye FM4-64 (ref: T13320; Thermo Fisher Scientific) was directly added to the medium 30 min before imaging at a final concentration of 5 μM (1:2,000 dilution from a 5 mg/ml stock in DMSO). A Nikon CSU-W1 SoRa microscope with controlled temperature at 18°C was used for live-cell imaging. Coenocytes were imaged with a 60× water immersion objective (SR P-Apochromat IR AC 60× WI/ 1.27), and a TRITC emission laser was used at 5% laser power with 50-ms exposure time using a z-step size of 0.8 μm, 40 slices, and 10-min time intervals.

### TEM tomography and tilt series reconstruction

For sample preparation for electron microscopy, coenocytes were diluted 1:50 in 1/16 MB and grown for 5 d to obtain saturated cultures. The saturated cultures were then diluted 1:70 in fresh cold MB (Falcon 75-cm^2^ Rectangular Canted Neck Cell Culture Flask with Vented Cap, product number 353136; Corning) and grown for 28 h. A volume of 20 ml per time point per condition was used. To increase synchronization, coenocytes were scraped from flasks, collected by centrifugation at 500 rpm at 17°C, and washed three times in seawater to remove all the nutrients from the medium. Culture flasks were also washed with seawater, and cell suspensions in seawater were distributed with same initial volume into the culture flasks. Coenocytes were maintained in seawater for 8 h and to enrich for cellularization events, and treatment with DMSO (1:100) and MBC (2.5 mg/ml stock in DMSO, final concentration 25 μl/ml) was performed in the last 2 h before high-pressure freezing. High-pressure freezing was done at time points 36 and 36.5 h hpi. *S. arctica* cells were concentrated by centrifugation at 500 rpm for 5 min at 17°C and high-pressure–frozen with the HPM010 (Abra Fluid) using 200-μm-deep, 3-mm-wide aluminum planchettes (Wohlwend GmbH). Freeze substitution was done using the AFS2 machine (Leica Microsystems) in a cocktail containing 1% OsO4, 0.1% uranyl acetate, and 5% water in acetone. The samples were incubated as follows: 73 h at −90°C, temperature increased to −30°C at a rate of 5°C /h; 5 h at −30°C, temperature increased to 0°C at a rate of 5°C/h; 4 × 0.5-h rinses in water-free acetone at 0°C. This was followed by Spurr resin infiltration (25%—3 h at 0°C, 50% overnight at 0°C; 50%—4 h at room temperature; 75%—4 h, 75% overnight, 100% 4 h [×2], and 100% overnight). This was followed by exchange with 100% Spurr resin (4 h × 2, overnight). After this, the samples were polymerized in the oven at 60°C for over 2 d. The samples were then cut using an ultramicrotome (Leica UC7) into 70-nm sections and screened by 2D TEM (JEOL 1400 Flash) to assess sample preparation. For TEM tomography, 300-nm sections were poststained with 2% uranyl acetate in 70% methanol (5 min, room temperature) and in Reynolds’ lead citrate (2 min, room temperature). The tomograms were imaged on a Tecnai F30 microscope using SerialEM and reconstructed using iMOD/eTOMO (38, 39).

### FIB-SEM and segmentation

The Spurr resin–embedded samples were prepared as described above. 70-nm sections were collected and screened by 2D TEM (JEOL 1400 Flash) to target cellularizing cells for FIB-SEM imaging as previously described (28). The samples were then mounted on a SEM stub using silver conductive epoxy resin (Ted Pella), gold sputter–coated (Quorum Q150RS), and imaged by FIB-SEM. The acquisition was performed using a Crossbeam 540 (Zeiss) following the Atlas 3D Nanotomography workflow. SEM imaging was done with an acceleration voltage of 1.5 kV and a current of 700 pA using an EsB detector (1,100 V grid). Images were acquired at 8 × 8 nm^2^ pixel size, and 8-nm slices were removed at each imaging cycle. FIB milling was performed at 1.5 nA current. For image analysis, we binned the dataset to a final voxel size of 16 × 16 × 16 nm^3^. Segmentation and visualization were done with Amira (v.2019.3 or 2020.1; Thermo Fisher Scientific).

### Image analysis, quantification, and statistical analysis

#### Analysis of time-lapse movies with FM4-64 dye

To measure cell diameter and PM furrow length in Figs 1, 3, and 4, we cropped time-lapse movies of FM4-64–labeled coenocytes to have a single coenocyte per movie. For each cropped time lapse, the middle section was used for all the measurements. The line tool from Fiji was used to measure the diameter and PM furrow length manually, for 3–4 PM furrows per coenocyte per treatment (40, 41). Movies in which invagination had already begun at the first imaged frame were excluded, because all timings are referenced to the onset of invagination rather than to the start of acquisition. Nuclei are visible in these movies as regions from which FM4-64 is excluded and are scored on single optical sections at both low and high display contrast. To calculate the internuclear distance, 0-min time point was selected. Distance was measured from the center of the nucleus to the center of their two nearest neighbors. Distances were measured for all visible nuclei in the middle section and averaged to obtain internuclear distance/coenocyte. To calculate PM furrow spacing, we selected the time point of 10 min in which PM furrows start to ingress at a value of 2–5 μm. Then, a circle line equal to the coenocyte diameter was positioned at the cortex of each coenocyte and defined with a width of 10. Using the plugin “area to line” to create a plot profile with the signal intensity for each furrow, furrow spacing was calculated as the distance between each peak of the plot profile. Furrow ingression rate was calculated as the slope of a linear fit to each furrow’s time points before it reached its maximum tracked length, thereby excluding the subsequent plateau. Furrows tracked at fewer than three time points were excluded, and the same criterion was applied to the maximum length reached by each furrow. Tracking ended although furrows were still elongating in almost every coenocyte, so this value is the length reached during tracking and not a final furrow length. The duration of cellularization was measured from the onset of membrane invagination to Flip, the point at which the coenocyte reorganizes and newborn cells become distinguishable. Timing is referenced to the onset of invagination rather than to the first imaged frame, because acquisition began at an arbitrary point in each coenocyte’s progression. The 10-min acquisition interval sets the precision at one interval per coenocyte. The invagination angle was measured on a single midsection as the angle between the axis of an invagination and the local cortex, for 10 furrows per coenocyte at matched ingression depth between conditions. It is reported as the deviation from perpendicular, the absolute difference from 90 degrees, because furrows in treated coenocytes deviate in both directions and the raw angle therefore averages close to the control value. Released cell area was segmented from live FM4-64 after cellularization, taking 8–12 released cells per coenocyte, and is reported as the coefficient of variation of area within each coenocyte. Cell perimeter was calculated in a semi-automated manner by first subtracting the background (50 pixels) of each cropped time-lapse movie and applying a Gaussian blur filter (sigma = 1). Using an Otsu threshold, a black-and-white mask was created, and cell perimeter was then measured using the Wand tool and the “measure” function in Fiji.

#### From U-ExM images

Image analysis (Fig 2) was done using Fiji (40, 41). Coenocyte diameter/radius, MT length, nuclear distance from the cortex, and PM furrow length from U-ExM images were measured by performing maximum-intensity projections of 15–20 sections from the center of the coenocyte. Using the line tool from Fiji, four different compartments or nuclei per coenocyte were quantified. MT length (tubulin channel) was measured from the MTOC toward the center of the coenocyte. Nuclear distance from the cortex was calculated as the Euclidean distance between the nucleus and the cortical membrane using the DNA channel (Hoechst) and membrane channel (BODIPY) to find the edges of cells in the coenocyte. MT lengths in centrifuged coenocytes were measured differently, on maximum-intensity projections of 15 consecutive z-sections centered on the equatorial plane and applied identically to every coenocyte and to the uncentrifuged controls. For each nucleus, the longest MT of its aster was measured in any direction, from the MTOC to the distal tip, and corrected for the expansion factor. Nuclei were counted by scrolling the stack rather than on the projection, because nuclei at different depths overlap in projection. Tubulin-positive objects in the nuclear-depleted cortex were recorded separately from nuclear asters.

#### Region-paired measurements in centrifuged coenocytes

Centrifugal displacement was performed as described previously (26). Coenocytes pregrown for 36 h were centrifuged, using an Eppendorf 5425/5430 microcentrifuge with an FA-45-30-11 fixed-angle rotor, for 5 min either at 500 rpm, a low-speed control matched to the sedimented condition of that study, or at 12,000 rpm to displace nuclei, after which live imaging was performed with or without small molecule inhibitors as indicated above. As displacement is partially penetrant under these conditions, analyses were therefore restricted to coenocytes showing clear nuclear displacement (26). Within each centrifuged coenocyte, a nuclear-enriched and a nuclear-depleted region were identified by eye on a single midsection, and only regions that could be classified confidently were measured. All measurements were made 10 min after the onset of invagination. Furrow spacing, furrow length, and invagination angle were measured as described above, up to eight measurements per region and fewer where fewer were resolvable in that plane; this count is set by the measuring protocol and is not a furrow count. These measurements were made on furrows that had progressed far enough to be traced unambiguously in the chosen section. Nuclear-depleted regions carry many additional small indentations at the cortex that do not sustain ingression; these were not traced and are absent from the values reported.

#### Statistical analysis

The coenocyte is the replicate unit throughout. Furrows, interfurrow gaps, nuclear pairs, and released cells measured within one coenocyte are not independent of one another and were summarized to a single value per coenocyte before any test. Quantities were summarized by their mean, except interfurrow spacing, which is right-skewed within a coenocyte, is positive in 86% of coenocytes, and is summarized by its median. Dispersion is reported as the coefficient of variation, sd over mean. It stays comparable when the means themselves differ. Concretely, two independent groups were compared with a Mann–Whitney U test, the two regions of one centrifuged coenocyte were compared with a paired Wilcoxon signed-rank test, and correlations were done by Spearman’s rank test. Planned comparisons were corrected within a declared family by the Benjamini–Hochberg test, and families were never pooled. Every panel carries its exact p rather than a significance class. This matters at these sample sizes, because rank tests are discrete as a paired Wilcoxon test at n = 5 cannot return below 0.0625, and a Mann–Whitney test at 4 versus 3 cannot return below 0.057. Panels sitting at that limit say so in their legend, where a value above 0.05 marks complete separation rather than no difference. Statistics were computed in R, and the underlying per-coenocyte values are provided in SourceData for Figures.

RStudio (R software) was used to plot PM furrow length over time, and to change the aesthetics of the plots. Plots were generated in RStudio. All Figs were assembled with Adobe Illustrator.

### Use of generative AI

Claude Opus (Anthropic) was used as a coding and editing assistant during the final revision of this article. It assisted in spelling, grammar, and refactoring the R scripts for figure panels. All experiments, image acquisition, image analysis, and quantification were performed by the authors. Every script, plot, statistical output, and passage of text produced with this assistance was run, inspected, and verified by the authors, who take full responsibility for the content of this article. Claude is not an author and made no contribution meeting authorship criteria.

## Supplementary Material

Reviewer comments

## Data Availability

All image data related to this study are deposited at BioImage Archive (42). All quantification presented in the Figures is shared as SourceData for Figures.
