# Supplementary material for: Microtubules coordinate cellularization in a coenocytic relative of animals
Source: Life Sci Alliance. 2026 Oct 26;10(1):e202603917. doi: 10.26508/lsa.202603917 (PMC13710129; doi:10.26508/lsa.202603917)
Supplement: Supplementary file 1 [file LSA-2026-03917_review_history.pdf]

Microtubules coordinate cellularization in a coenocytic relative of animals

Margarida Araújo, Marine Olivetta, Paolo Ronchi, Viola Oorschot, Arif Khan, Christian Tischer, Hiral Shah, Gautam Dey, and Omaya Dudin
DOI: https://doi.org/10.26508/lsa.202603917

Corresponding author(s): Omaya Dudin, University of Geneva and Gautam Dey, European Molecular Biology Laboratory

Review Timeline:

Submission Date:	2026-09-24
Editorial Decision:	2026-10-07
Revision Received:	2026-10-10
Accepted:	2026-10-12

Scientific Editor: Tim Fessenden

Transaction Report:

(Note: With the exception of the correction of typographical or spelling errors that could be a source of ambiguity, letters and reports are not edited. The original formatting of letters and referee reports may not be reflected in this compilation.)

Please note that the manuscript was reviewed at *Review Commons* and these reports were taken into account in the decision-making process at *Life Science Alliance.*

Reviews

Review #1

1. Evidence, reproducibility and clarity:

The last several years have seen major advances in our understanding of the basic cell biology of the set of single cell animal relatives, led by the authors and their colleagues. These groups have developed several as models and pioneered remarkable microscopy approaches to examine their cell biology. Here the authors extend this work to the ichthyosporean Sphaeroforma arctica, exploring the fascinating process by which a syncytial life stage cellularizes, and seeking to define the role of microtubules and Golgi trafficking. Their new expansion microscopy delivers impressive images of membrane and the cytoskeleton during this process and has the promise of answering important questions about the roles of microtubules and membrane trafficking. I thus went into my review quite excited. However, as I detail below, while some aspects of the process are carefully quantified, the current manuscript draws multiple broad conclusions that do not seem fully supported by the limited data provided. This substantially reduced my enthusiasm. I'd also note, though I did not take this into account in my evaluation, that many of the images are presented at a size that was difficult to interpret, without my electronically enlarging them, and two of the Figures were mis-labeled.

1. Interpreting most of their Figures requires understanding the basics of cellularization in this organism. Comparing the diagram in Fig. 1B and the images in 1C left me confused. First, are all the images in 1C and similar images later cross sections? Are nuclei dispersed throughout the cytoplasm at the start or restricted to a region near the cortex. If the former, how do more central nuclei get cellularized? The transition from the unperturbed 20 and 40 minute timepoints left me unclear on the normal process. Panel G may have been helpful in this regard but only shows the treated embryo and no untreated one.

2. The authors make a number of conclusions in Figure 1. Some are carefully quantified but others are not. For example, they state "producing uncoordinated ingression with variable rates, diagonal trajectories, and occasional bifurcations, in contrast to the uniform, perpendicular furrows observed in controls". I was not convinced by the single images provided that these were different-for example, spacing in the unperturbed 10 minute time point seems variable and furrows are not "uniformly perpendicular" in the unperturbed 20 minute time point. I also was puzzled by the lack of change in nuclear spacing while furrow spacing was altered. Later on in this section they state "MBC-treated coenocytes displayed significantly decreased and irregular furrow spacing, resulting in some compartments lacking nuclei entirely (Figure 1G & H)", but none of these images visualize nuclei directly-Perhaps the lower level background in H is supposed to indicate this but no parallel wildtype image is shown. Finally, while their TEM images (in the panels that are either I or J due to mislabeling) are lovely, I was not sure what to conclude from them- only the unperturbed images show the embryo surface for orientation and the lefthand unperturbed image is not as straight as they suggest-I certainly don't think these few images support their strong, detailed conclusions here: "MBC treatment resulted in aberrant membrane architecture, with furrows exhibiting bifurcated and convoluted morphology and abnormal fusion at the base of invagination, compared to the smooth, organized structure of control furrows".

3. The images in Fig. 2B are remarkable and very informative, though as I note above they are presented at such a small size that they require considerable enlargement to appreciate. The surprising accumulation of actin at the invagination front, presumably long before membrane closure begins, was striking, as were the microtubule baskets. However, conclusions drawn again seemed too strong. The authors state "High magnification views further supported that these bundles closely tracked the advancing furrow fronts, with longer MT extensions associated with deeper furrows during later stages (Figure 2D, arrowheads)" (BTW once again this Figure was not labeled in parallel with the text-should be 2C). I did not think the NHS staining provided sufficient resolution of advancing furrow fronts to draw this conclusion. They end this section with some more detailed conclusions, which did not seem to me to be well supported by the single image shown: "Furrows were often misaligned, and compartments frequently enclosed multiple nuclei or, conversely, lacked nuclei entirely. In several cases, nuclei were observed trailing between furrows or located beneath partially formed compartments, suggesting that improper nuclear positioning may interfere with furrow progression and sealing (Figure 2F)." The latter conclusion also seemed to leave me wondering about cause and effect. The final sweeping conclusions in the paragraph on p. 7 top (next time please include page numbers) thus seemed much too broad.

4. I thought the use of centrifugation to move nuclei was clever. However, it also is moving many other things-for example it moves whatever organelles are labeled by BODIPY and apparently nuclear associated MTOCs, leading to some caveats and calling into question their claim that it "doesn't disrupt MTs". Once again, broad conclusions were

drawn based on an n=1 image: "furrow ingression proceeded with kinetics comparable to controls, confirming that the core machinery for membrane trafficking and actin-driven invagination remained functional (Figure 3B). However, furrow spatial patterning was dramatically altered: in nuclear-depleted cortical regions, furrow initiations were more frequent and closely clustered, but failed to progress deeply. In nuclear-enriched regions, furrows progressed with kinetics comparable to controls but were misaligned, frequently enclosing multiple nuclei per compartment rather than the single nucleus observed in controls" Only one thing was quantified-furrow ingression rate-and this must have been done on the selected set of furrows that progressed, and not, for example, of ones like those at the bottom of the image series presented. None of the other conclusions about spatial patterning were quantified-for example, nuclei are not even visualized in Fig 3C. Finally, they do not even mention the results of the MT perturbation presented in this Figure, and the fact that few differences are apparent calls into question their conclusion that that "nuclei (and their associated MTOCs) serve as spatial landmarks that pattern membrane invagination".

5. 1. Figure 4 is surprisingly described in a single short paragraph. While some things were quantified, I was not convinced that they could conclude that they observed furrows "mispositioned like those seen upon MT depolymerization". More broadly, what do we really learn from this?

Referees cross-commenting

Unfortunately I remain convinced that this manuscript has significant issues with the match between the data presented and the claims made--I laid these issues out clearly and stand by them

2. Significance:

As I note in detail in the previous section, Here the authors extend this work to the ichthyosporean Sphaeroforma arctica, exploring the fascinating process by which a syncytial life stage cellularizes, and seeking to define the role of microtubules and Golgi trafficking. Their new expansion microscopy delivers impressive images of membrane and the cytoskeleton during this process and has the promise of answering important questions about the roles of microtubules and membrane trafficking. I thus went into my review quite excited. However, as I detail below, while some aspects of the process are carefully quantified, the current manuscript draws multiple broad conclusions that do not seem fully supported by the limited data provided. This substantially reduced my enthusiasm.

3. How much time do you estimate the authors will need to complete the suggested revisions:

Estimated time to Complete Revisions (Required)

(Decision Recommendation)

Cannot tell / Not applicable

4. Review Commons values the work of reviewers and encourages them to get credit for their work. Select 'Yes' below to register your reviewing activity at Web of Science Reviewer Recognition Service (formerly Publons); note that the content of your review will not be visible on Web of Science.

Web of Science Reviewer Recognition

No

Review #2

1. Evidence, reproducibility and clarity:

Summary

This interesting paper is a follow-up from Dudin et al.'s seminal 2019 eLife paper describing cellularization in the ichthyosporean Sphaeroforma arctica. In that earlier story, a role for microtubules (MTs) in cellularization was supported by treatment with the MT-depolymerizing drug MBC, which deeply affected nuclear spacing and the regularity of cellularization. However, the difficulty of imaging microtubules at the time had prevented more in-depth functional studies. This technical barrier has now been lifted by ultrastructural expansion microscopy (U-ExM), and this paper thus picks up where the earlier study left off.

The study combines live imaging, drug treatments, electron microscopy and ultrastructural electron microscopy to support a role for microtubules, nuclei, and membrane trafficking in sustaining the fidelity of S. arctica cellularization. An extensive live imaging dataset and careful image quantifications reinforce and expand the previously published

observation that microtubules, while dispensable for cellularization to occur at all, are necessary for it to occur with proper timing and spacing. UEx-M images support the idea that actin and microtubules guide plasma membrane invaginations, and centrifugation experiments support a role for nuclear positioning in ensuring fidelity of cellularization. Finally, Brefeldin A treatment followed by live imaging and U-ExM supports a role for membrane trafficking in furrow positioning and elongation.

Major comments

The conclusions are adequately supported by the data, and their limitations are transparently acknowledged: notably, it is not fully clear by what mechanisms nuclei guide cellularization, and whether those mechanisms depend on microtubules or not. Below are a few points where I feel additional data (or better visualization, or additional verbal caveats) could improve the manuscript.

1) After 12,000rpm centrifugation, the authors point out that furrows "were misaligned, frequently enclosing multiple nuclei per compartment". This is not obvious in Figure 3C, where nuclei are only visible as empty spaces and only labelled (by asterisks) at a relatively early stage of furrow ingression. This point could be better supported by more explicit images (with nuclei more evident at late stages, even if only as blank spaces), or maybe by co-staining DNA and either membrane or F-actin in centrifuged samples.

2) In centrifuged samples (Figure 3A), some staining is visible in tubulin channel of the nuclei-free part, but does not correspond to discrete, observable microtubules. Could the authors comment on this? Do they think these represent diffuse, perhaps damaged microtubules (nucleated independently of nuclei?), or perhaps free tubulin, or mere background?

3) Similarly: in centrifuged samples Fig. 3C, the nuclei-free part of the cell does seem to cellularize, albeit slower and forming smaller compartments than the nucleated part. This suggests that nuclei (and microtubules?) guide cellularization, but are perhaps not necessary for it. Could the authors comment?

4) The bottom row of Figure 3C presents a series of experiments combining MBC treatment with 12,000rpm centrifugation, but if I am not mistaken these are not discussed at all in the text. This is unfortunate, as it would be interesting to know what the authors want to conclude from these experiments (whose results are however not quantified, perhaps limiting their scope). In my view, one reason to be interested in this dataset is that it could in principle inform epistatic relationships between nuclei positioning and microtubules, that are otherwise left open: do these guide furrow ingression independently of each others (in which case their effects should be additive) or does one act purely through the other (in which case the combined treatment should not be worse than individual treatments)? In any case, I would suggest either commenting on these data explicitly (perhaps with additional quantification guiding interpretations) or removing them.

Minor comments

- Figure 1 contains two distinct panel H's.

- Typo p. 6: "actomyosing"

- Page 7: "longer MT extensions associated with deeper furrows during later stages (Figure 2D, arrowheads)" rather seems to refer to Figure 2B or C.

- Line and page numbers are missing

Referees cross-commenting

After reading through the two other reviewer comments, I feel we are in substantial agreement that (1) the paper deals with an interesting issue and has potential; (2) it can be substantially improved by better quantifications and better visualization of nuclei across several experiments, which would be key to support some of the conclusions. Given the relatively short life cycle of S. arctica, I still believe that revisions satisfying all reviewers should be achievable within a reasonable time frame - I would suggest 1 to 3 months.

2. Significance:

This paper will be of broad interest to readers interested in the evolution of multinucleated cells and cellularization, and the recurrent involvement of actin and microtubules in these processes (see notably recent papers by the Brugués lab). While it could appear incremental in an ichthyosporean-centric perspective (notably compared to the 2019 eLife paper that had anticipated some of the conclusions), its comparative implications gives it an additional

scope in my view. Perhaps this is something the authors themselves could emphasize a bit more in their own conclusion. Finally, although most techniques had been established in earlier papers by the same authors, this study further confirms their robustness and the power of S. arctica as an emerging cell biology model - another nice touch.

3. How much time do you estimate the authors will need to complete the suggested revisions:

Estimated time to Complete Revisions (Required)

(Decision Recommendation)

Less than 1 month

4. Review Commons values the work of reviewers and encourages them to get credit for their work. Select 'Yes' below to register your reviewing activity at Web of Science Reviewer Recognition Service (formerly Publons); note that the content of your review will not be visible on Web of Science.

Web of Science Reviewer Recognition

Yes

Review #3

1. Evidence, reproducibility and clarity:

In their manuscript, Araújo et al. described their investigation into the cellularisation process in Sphaeroforma artica during the multicellular stage of its life cycle. The coenocytes are relatively small at 50 µm in diameter, but the authors developed expansion microscopy and used a new actin probe to provide a detailed description of the ingression of the membrane furrow between adjacent nuclei. They compared the formation of the furrow in the presence or absence of microtubules, as well as in conditions involving regularly spaced or clustered nuclei. They concluded that, while microtubules were not essential to the formation and ingression of the furrow, they were required for the fidelity of the process, as stated in the title of the manuscript.

A better quantification of the ingression pattern would have improved the data. The centrifugation experiment is particularly interesting, as it forces the accumulation of nuclei on one side of the coenocytes. Surprisingly, this only partially perturbs the position of the furrow. This showed, on the one hand, that nuclei contribute to the positioning of the furrow close to them, and, on the other hand, that an additional mechanism exists independently of them. The analysis does not clarify whether the absence of microtubules in these centrifuged states affects the position of the furrow. Figure 3C seems to suggest partial rescue (suggesting that the contribution of nuclei is microtubule-dependent, but that the peripheral membrane has its own partitioning mechanism), but this has not been quantified.

One minor concern is that it remains unclear whether microtubule disruption affects the positioning of the nuclei, as stated in the text but not confirmed by the quantification (Figure 1F).

2. Significance:

The work is primarily descriptive, and the processes determining the position of the furrow and driving its ingression remain unknown. Nevertheless, the work provides beautiful images of a poorly described yet potentially informative system. These are distant relatives of animals with interesting common characteristics, such as the cellularisation process. The conservation of this process may reveal some key fundamental properties of multicellularity. Despite its limited conceptual advances, the manuscript is thus innovative and interesting.

3. How much time do you estimate the authors will need to complete the suggested revisions:

Estimated time to Complete Revisions (Required)

(Decision Recommendation)

Less than 1 month

4. Review Commons values the work of reviewers and encourages them to get credit for their work. Select 'Yes' below to register your reviewing activity at Web of Science Reviewer Recognition Service (formerly Publons); note that the content of your review will not be visible on Web of Science.

Web of Science Reviewer Recognition

Yes

RESPONSE TO REFEREES

We sincerely appreciate your time, effort and expertise. Your comments were insightful and constructive, and over the last months, we have made it our main priority to fully address your comments and concerns. The revised paper is, in our opinion, far stronger as a result of peer review. Thank you.
Throughout this response document, we use **blue text** to indicate our response, **black text to indicate the referee's original comment**, and **magenta** text to indicate a quote taken from the manuscript.

Reviewer #1
Evidence, reproducibility and clarity
The last several years have seen major advances in our understanding of the basic cell biology of the set of single cell animal relatives, led by the authors and their colleagues. These groups have developed several as models and pioneered remarkable microscopy approaches to examine their cell biology. Here the authors extend this work to the ichthyosporean Sphaeroforma arctica, exploring the fascinating process by which a syncytial life stage cellularizes, and seeking to define the role of microtubules and Golgi trafficking. Their new expansion microscopy delivers impressive images of membrane and the cytoskeleton during this process and has the promise of answering important questions about the roles of microtubules and membrane trafficking. I thus went into my review quite excited. However, as I detail below, while some aspects of the process are carefully quantified, the current manuscript draws multiple broad conclusions that do not seem fully supported by the limited data provided. This substantially reduced my enthusiasm. I'd also note, though I did not take this into account in my evaluation, that many of the images are presented at a size that was difficult to interpret, without my electronically enlarging them, and two of the Figures were mis-labeled.

The general points raised here are dealt with below. All panels in the main figures are enlarged, zoom-ins are added where they help, and figures that are no longer fitted have been moved to the supplementary figures. Panel labelling has been checked throughout, the two mislabelled figures are corrected, and the labelling is now consistent across the whole figure set. The additional quantification and the associated framework is set out under comment 2.

1. Interpreting most of their Figures requires understanding the basics of cellularization in this organism. Comparing the diagram in Fig. 1B and the images in 1C left me confused. First, are all the images in 1C and similar images later cross sections? Are nuclei dispersed throughout the cytoplasm at the start or restricted to a region near the cortex. If the former, how do more central nuclei get cellularized? The transition from the unperturbed 20 and 40 minute timepoints left me unclear on the normal process. Panel G may have been helpful in this regard but only shows the treated embryo and no untreated one.

This comment identified a real shortcoming in how we presented the system, and it prompted the largest single change to the text of the revised manuscript. In writing the

original version we took the basic description of cellularization in *S. arctica* largely for granted, since it has been built up across several previous studies (Dudin et al. 2019, Ondracka et al. 2018, Olivetta et al. 2023 and Shah et al. 2024), and we did not restate it for a reader arriving at the organism for the first time. We have addressed this on three fronts: the imaging planes, the referee's question about nuclei, and the introduction.

On the images themselves (Figure 1), these panels and the equivalent images throughout the manuscript are equatorial mid-sections. The imaging plane is now explicitly stated in every figure legend, so that single slices, equatorial mid-sections, and maximum-intensity projections can no longer be confused with one another. More usefully, we have added Figure EV1A, which shows an untreated cellularizing coenocyte as both a top view and an equatorial mid-section, at the onset of invagination and 10 minutes later, each at low and high contrast. Figure EV1A makes the two geometries directly comparable, rather than leaving the reader to reconcile the schematic in Figure 1C with a single plane, and it also provides the untreated counterpart to Figure 1G that the referee requested at the end of this comment. All parameters of cellularization measure in the study are now indicated on the sketch in Figure 1C.

Figure Legend EV1A: (A) Cellularizing control *S. arctica* coenocytes labelled with FM4-64, shown as a top view and an equatorial mid-section at the onset of invagination (0 min) and 10 min later, each at low and high contrast display. Nuclei are visible as dye-excluded regions (asterisks). Scale bar, 10 µm.

We would add that an untreated comparison was in fact already available, since Movie EV1 runs a control and an MBC-treated coenocyte side by side through the whole of cellularization at 10-minute intervals. We accept that this was not where the referee would naturally have looked for it, and Figure EV1A now places the comparison in the figures themselves.
On the referee's central question, we can be unambiguous. Nuclei are not dispersed throughout the cytoplasm From the coenocytic phase through to cellularization, they remain associated with the cortex, and this has been established consistently across the previous work cited above. There are therefore no central nuclei in controls, and the question of how they would be enclosed does not arise. We recognize, however, that the original manuscript never stated this outright, and that a reader had no way of knowing it, and the referee was right to ask. The revised text now says it explicitly and follows it with the geometric consequence.

Line 70-72: Nuclei divide synchronously and rather than occupying the interior of the coenocyte, associate with the cortex, evenly spacing out along the surface.

Line 78-82: Recent work has shown that *S. arctica* cellularization is regulated by the nucleocytoplasmic (N/C) ratio, with cellularization timing tightly coupled to nuclear content relative to cytoplasmic volume. Invaginations initiate at the cell periphery, between adjacent cortical nuclei, and advance inward, carving compartments from the cortex around each nucleus rather than assembling them from the cell interior.

Finally, the introduction has been substantially expanded so that the process is defined prior to the figures. It now sets out the coenocytic phase, the arrangement of nuclei at the cortex, the direction in which invaginations progress, and the stages through to the end of cellularization, drawing explicitly on the previous characterisations that the original manuscript had assumed the reader would already know. This also addresses the referee's difficulty with the transition between the unperturbed timepoints, which we had described without first establishing what the normal sequence is.

2. The authors make a number of conclusions in Figure 1. Some are carefully quantified but others are not. For example, they state "producing uncoordinated ingression with variable rates, diagonal trajectories, and occasional bifurcations, in contrast to the uniform, perpendicular furrows observed in controls". I was not convinced by the single images provided that these were different-for example, spacing in the unperturbed 10 minute time point seems variable and furrows are not "uniformly perpendicular" in the unperturbed 20 minute time point. I also was puzzled by the lack of change in nuclear spacing while furrow spacing was altered. Later on in this section they state "MBC-treated coenocytes displayed significantly decreased and irregular furrow spacing, resulting in some compartments lacking nuclei entirely (Figure 1G & H)", but none of these images visualize nuclei directly-Perhaps the lower level background in H is supposed to indicate this but no parallel wildtype image is shown. Finally, while their TEM images (in the panels that are either I or J due to mislabeling) are lovely, I was not sure what to conclude from them- only the unperturbed images show the embryo surface for orientation and the lefthand unperturbed image is not as straight as they suggest-I certainly don't think these few images support their strong, detailed conclusions here: "MBC treatment resulted in aberrant membrane architecture, with furrows exhibiting bifurcated and convoluted morphology and abnormal fusion at the base of invagination, compared to the smooth, organized structure of control furrows".

We have considerably expanded both the range and the amount of quantification in the revised manuscript, and every quantity we measure is now sketched out in Figure 1C. These measurements are organised around the four properties of cellularization that the revised manuscript is built on:

1. Nuclear organisation, measured as inter-nuclear distance and its variability within a coenocyte (Figure EV1G and Figure 1F).
2. Furrow positioning, measured as furrow spacing, its variability within a coenocyte, and the deviation of the invagination axis from perpendicular to the cortex (Figure 1H, Figure EV1H and Figure EV1I).
3. Furrow ingression dynamics, measured as ingression rate, its variability, the maximum length furrows reach, its variability, and the duration of cellularization from the onset of invagination to Flip (Figure 1E and Figure EV1B-F).
4. Developmental outcome, measured as the variability of released cell size (Figure EV1J).

The same set of measurements is now applied to every perturbation in the manuscript, so that microtubule depolymerization, centrifugal displacement of nuclei and disruption of membrane trafficking are each characterised against the same quantities rather than

described in their own terms. Applied across the whole dataset, these measurements answer the referee's objections directly.
The descriptors they quote were asserted from single images rather than measured. We have deleted "diagonal trajectories", "occasional bifurcations" and "uniform, perpendicular" from the manuscript, and what remains is carried by measurement.

Line 113-115: MT loss disrupted furrow ingression dynamics, producing uncoordinated ingression - in contrast to the consistent furrows observed in controls (Fig. 1D, Movie EV1).

Coordination is now measured within single coenocytes, so that furrows are compared against their own neighbours rather than across the population. This is the comparison the referee implies when looking at a single image and asking whether the furrows within it differ. It is now Figure EV1D.

Figure Legend EV1D: (C) Variability of furrow ingression rate within a coenocyte, from the rates in (B). DMSO 0.053, MBC 0.111, p = 0.014.

Perpendicularity is measured in Figure EV1L, on ten furrows per coenocyte at matched ingression depth between conditions. This concedes the referee's specific point: control furrows are not uniformly perpendicular, and deviate by 5.51 degrees on average. We claim only that treated coenocytes deviate roughly twice as far.

Figure Legend EV1I: (I) Deviation of the invagination axis from perpendicular to the cortex, |angle - 90|, averaged per coenocyte. DMSO 5.51 degrees (n=5), MBC 10.83 degrees (n=4), Ten furrows per coenocyte; p = 0.016. Ingression depth did not differ between conditions.

On the referee's puzzlement about nuclear spacing, they have identified something we had underplayed. Mean inter-nuclear spacing genuinely does not change, and we do not claim that it does. What changes is its regularity, which roughly doubles. The same pattern recurs for furrow spacing, for the length furrows reach, for ingression rate and for the size of the cells finally released, and it is why the manuscript always argued that microtubules sustain the fidelity of cellularization rather than its execution. However, now it's strengthened by numerical data.

Line 156-159: While mean inter-nuclear spacing was not significantly different between conditions (6.15 *vs* 6.22 µm, Fig. EV1G), MBC-treated coenocytes exhibited dramatically increased variability in nuclear positioning (0.143 *vs* 0.268, Fig. 1F), with nuclei ranging from properly positioned to severely mislocalized.

On the visualisation of nuclei, we would push back in part. Nuclei are directly visible in FM4-64 as regions from which the dye is excluded, and this is how they were scored throughout; the approach is not introduced here and was used in this same organism in Olivetta and Dudin (2023). We accept that this was never explained and that the display contrast made it difficult to see. The Methods now state the scoring explicitly; Figure EV1A

and Figure EV3D show coenocytes at both low and high display contrast, and nuclei are marked with asterisks.

Figure legend EV1: (A) Cellularizing control *S. arctica* coenocytes labelled with FM4-64, shown as a top view and an equatorial mid-section at the onset of invagination (0 min) and 10 min later, each at low and high contrast display. Nuclei are visible as dye-excluded regions (asterisks). Scale bar, 10 µm.

Line 272-274: Throughout these experiments nuclei are resolved in single optical sections as regions from which FM4-64 is excluded and are visible as such at both low and high display contrast (Fig. EV3D).

The claim about compartment contents has also been moved to data where nuclei are directly labelled rather than inferred. The statement that some compartments lacked nuclei is removed from the furrow spacing sentence, and the observation is now made on U-ExM with a DNA stain, in Figure EV2C and Movie EV5, where it is made on a single section and on the full volume rather than on a projection.

Figure EV2C: (C) Two MBC-treated coenocytes: a maximum intensity projection of 50 consecutive z-sections (left) and a single z-section of a second coenocyte (right). Tubulin signals persist around a subset of nuclei after MT depolymerization, and individual compartments enclose more than one nucleus. This observation is made on the single section and on the full volume in Movie EV5, not on the projection, since nuclei at different depths overlap in projection. Scale bar, 10 µm. Scale bars are adjusted for expansion factors.

The mislabelled panels have been corrected throughout, and we thank the referee for catching them.

TEM images of furrows with visible cell surface marked as yellow asterisks are now included in the main figure. Additional images of furrows (furrow 3 and 4) and zoom-ins of the convoluted membrane at the tip are now included in the supplementary Figure EV1K,L. These are representative images selected from DMSO (n = 25 furrows from 34 tomograms, with cell surface seen in 19), MBC (n = 67 furrows from 72 tomograms, with cell surface visible in 37), total 106 tomograms imaged across different cellularization stages and conditions. This information is now included in the legend of Figure 1J. TEM images of DMSO and MBC-treated full cells are provided in EV1K to provide an overview of furrow consistency in the two conditions. Nuclei are labelled with N to indicate nuclei per compartment.

3. The images in Fig. 2B are remarkable and very informative, though as I note above they are presented at such a small size that they require considerable enlargement to appreciate. The surprising accumulation of actin at the invagination front, presumably long before membrane closure begins, was striking, as were the microtubule baskets.

However, conclusions drawn again seemed too strong. The authors state "High magnification views further supported that these bundles closely tracked the advancing furrow fronts, with longer MT extensions associated with deeper furrows during later stages (Figure 2D, arrowheads)" (BTW once again this Figure was not labeled in parallel with the text-should be 2C). I did not think the NHS staining provided sufficient resolution of advancing furrow fronts to draw this conclusion. They end this section with some more detailed conclusions, which did not seem to me to be well supported by the single image shown: "Furrows were often misaligned, and compartments frequently enclosed multiple nuclei or, conversely, lacked nuclei entirely. In several cases, nuclei were observed trailing between furrows or located beneath partially formed compartments, suggesting that improper nuclear positioning may interfere with furrow progression and sealing (Figure 2F)." The latter conclusion also seemed to leave me wondering about cause and effect. The final sweeping conclusions in the paragraph on p. 7 top (next time please include page numbers) thus seemed much too broad.

The descriptive claim has been replaced by a measurement made across the whole dataset, and the causal claim has been removed altogether.
On presentation, all panels in Figure 2 are enlarged in the revised version. We have also added zoom-ins on a forming compartment in Figure EV2A, and a second late-stage example shown as single channels and merge in Figure EV2B, so that the relationship between the microtubule network and the invaginating membrane can be inspected at a useful magnification rather than inferred from a small panel.
The sentence the referee quotes has been deleted. Their objection is well founded, since we were reading furrow fronts off the pan-labelling and then drawing a quantitative conclusion about depth from it. In its place, the relationship between microtubule length and furrow depth is now measured directly and reported as a correlation across 68 coenocytes, and we state explicitly what that correlation does not establish.

Line 209-214: Quantitative analysis of MT networks showed that -MT length scaled with furrow depth (Spearman rho = 0.709, $p = 1.3 \times 10^{-11}$, n = 68 coenocytes), consistent with MTs elongating in coordination with plasma membrane invagination (Figs. 2E,F). While this correlation suggests a role for MTs in furrow progression, it remains unclear whether this involves active polymerization at the furrow front or utilization of pre-formed MT tracks.

On the second part quoted, the speculation that improper nuclear positioning may interfere with furrow progression and sealing has been removed. This addresses the point about cause and effect directly, since we cannot separate the two from these data and we no longer imply that we can. As set out in our response to comment 2, the claim that compartments lacked nuclei is also removed, and what remains of that observation now rests on Figure EV2C and Movie EV5 rather than on a single image, so we do not repeat it here.

Line 218-221: Furrows were often misaligned, and individual compartments were observed to enclose more than one nucleus (Fig. EV2C, Movie EV5). Nuclei were also

seen trailing between furrows or lying beneath partially formed compartments (Movie EV5).

The panel that should have been cited as Figure 2C is corrected, as part of the labelling pass described under comment 2, and the callouts have been checked so that each panel is cited in the order of its lettering. Line numbers are included in this revised version, as the referee requests.
Finally, in the closing paragraph of the section, we accept that it drew broader conclusions than the data in that section carried. It has been rewritten so that each claim is tied either to a specific measurement or to a named comparison with another system, and the section now ends on the comparison between our two titratable perturbations across ten measurements in Figure 4F rather than on a general statement about cytoskeletal coordination.

4. I thought the use of centrifugation to move nuclei was clever. However, it also is moving many other things-for example it moves whatever organelles are labeled by BODIPY and apparently nuclear associated MTOCs, leading to some caveats and calling into question their claim that it "doesn't disrupt MTs". Once again, broad conclusions were drawn based on an n=1 image: "furrow ingression proceeded with kinetics comparable to controls, confirming that the core machinery for membrane trafficking and actin-driven invagination remained functional (Figure 3B). However, furrow spatial patterning was dramatically altered: in nuclear-depleted cortical regions, furrow initiations were more frequent and closely clustered, but failed to progress deeply. In nuclear-enriched regions, furrows progressed with kinetics comparable to controls but were misaligned, frequently enclosing multiple nuclei per compartment rather than the single nucleus observed in controls" Only one thing was quantified-furrow ingression rate-and this must have been done on the selected set of furrows that progressed, and not, for example, of ones like those at the bottom of the image series presented. None of the other conclusions about spatial patterning were quantified-for example, nuclei are not even visualized in Fig 3C. Finally, they do not even mention the results of the MT perturbation presented in this Figure, and the fact that few differences are apparent calls into question their conclusion that that "nuclei (and their associated MTOCs) serve as spatial landmarks that pattern membrane invagination".

This comment is well taken on every count, and the centrifugation experiment has been reanalysed accordingly. Every claim in that section is now quantified. The comparisons are paired within single coenocytes (nuclei depleted vs enriched regions) so that each coenocyte serves as its own control. Moreover, the microtubule part of the experiment is analysed and reported rather than left aside. The new quantifications are the following:

1. Nuclear displacement itself, as the percentage of nuclei in the enriched region per coenocyte before and after centrifugation (Figure EV3B).
2. The state of the microtubule network after centrifugation, as the length of the longest microtubule of each nuclear aster (Figure EV3C).
3. Furrow spacing in the nuclei-enriched and nuclei-depleted regions of the same coenocyte and its variability (Figure 3E and Figure EV3F).
4. Furrow length by region and its ratio (Figure EV3G).

5. Ingression rate by region and across spin conditions, and the invagination angle by region (Figure 3C, Figure 3D and Figure EV3E).

On MTs , we agree that the original claim was not supportable and it has been removed. We now measure the network rather than assert that it is intact. Centrifugation does not leave MTs undisturbed. It relocates them together with the nuclei, and the same sentence reports what is lost from the depleted cortex.

Line 269-276: Microtubules were relocated with the nuclei as every nucleus retained the associated MTOC and MT network, and the length of the longest microtubule of each network was unchanged by centrifugation (5.97 against 5.08 µm, Fig. EV3C). Throughout these experiments nuclei are resolved in single optical sections as regions from which FM4-64 is excluded and are visible as such at both low and high display contrast (Fig. EV3D). In the nuclei-depleted cortex of the same coenocytes, tubulin was present only as some short fragments, a median of three per coenocyte with a median length of 0.95 µm (Data EV1).

On the broader point that centrifugation moves more than nuclei, the referee is right and we do not present it as a clean perturbation. The manuscript states that displacement is only partially penetrant (Olivetta et al. 2023), gives the previously reported figures for irregular invagination and lysis under identical conditions, and restricts the analysis to coenocytes with clear nuclear displacement.

Line 257-263: This approach builds on previous work where we used centrifugation to perturb the spatial relationship between nuclei and the cortex and demonstrate that cellularization in *S. arctica* is sensitive to local nucleocytoplasmic ratios.[26] Centrifugal displacement is partially penetrant, with approximately 40% of centrifuged coenocytes showing irregular plasma membrane invaginations and about 10% undergoing lysis.[26] Analyses were therefore restricted to coenocytes showing clear nuclear displacement.

The observation about selection is also correct, and we have made it explicit rather than leaving it implicit. Measurements were indeed made on furrows that progressed far enough to be traced, and the nuclei-depleted cortex carries many additional small indentations that never sustain ingression. We now state this in the Methods and in the legend of Figure 3C. The failure of those small indentations to progress is part of the phenotype itself.

Line 308-317: Furrow spatial patterning was dramatically altered: in nuclear-depleted cortical regions, furrow initiations were more frequent and closely clustered but failed to progress deeply (Figs. 3B and EV3D). Among the furrows that did progress, spacing was more variable in the nuclear-free region than in the nuclear-enriched half of the same coenocyte. (Fig. 3E). Since furrow spacing is reliably quantifiable only for furrows that progress, the effective furrow spacing in this nuclear depleted region appeared wider and was abolished upon MBC treatment (Fig. EV3F). Taking into account the high number of furrow initials in the nuclear-depleted region (Figs. 3B and EV3D), and furrow separation

in the region enriched with nuclei suggests a minimum furrow exclusion zone around individual nuclei and their MT networks.

On the visibility of nuclei, and as set out in our response to comment 2, nuclei are resolved in single optical sections as regions from which FM4-64 is excluded. In this figure they are now marked with asterisks * in the time-lapse panel, Figure 3B, and Figure EV3D shows a centrifuged coenocyte at both low and high display contrast, so we do not repeat the general point here.
The final point is the most important one, and the revised analysis answers it directly. The microtubule part is now reported, and far from showing few differences it reverses the relationship between nuclear position and furrow spacing. In control coenocytes the nuclei-depleted region carries wider gaps than the enriched one, and after MT depolymerization it carries narrower gaps.

Line 317-322: Furrows in nuclear-depleted regions reached shorter lengths (2.14 against 7.50 μm, Fig. EV3G), forming asymmetrically longer compartments on the nuclear-enriched side. This advantage in nuclear-enriched regions was lost upon MBC treatment confirming the role of nucleus-associated MT networks in maintaining the synchronous invagination and uniform cellular partitioning.

We have also weakened the conclusion the referee quotes, so that it claims a relationship rather than a mechanism.

Line 322-326: With centrifugation, though the nucleus-associated MT networks also migrate, the regular spacing of the cytoskeletal network at the cortex was disrupted, and furrows no longer exhibited the uniform spacing characteristic of control coenocytes, suggesting indeed that MT-defined nuclear territories serve as spatial landmarks that define the coordinates of membrane invagination.

5. Figure 4 is surprisingly described in a single short paragraph. While some things were quantified, I was not convinced that they could conclude that they observed furrows “mispositioned like those seen upon MT depolymerization”. More broadly, what do we really learn from this?

This section has been substantially expanded, and the comparison the referee doubted is now made statistically. The section now opens with what is known about membrane supply during cellularization in other systems, including the link between the Golgi and microtubules that makes this perturbation informative in the first place, and it reports a dose series rather than a single condition.

Line 363-375: While these experiments define how cellularization is spatially patterned, the cellular machinery driving membrane invagination itself remained to be identified. The previous experiments show that furrow invagination proceeds even when furrows are mispositioned. This indicates that the processes of new membrane addition and furrow positioning are controlled independently. To identify the cellular processes driving furrow

invagination, we examined the role of membrane trafficking. In early *Drosophila* embryos, the membrane expands from a reservoir of microvilli localized at the apical cortex.[33,34] The first phase of this membrane invagination is supplemented partly by Golgi-derived vesicles. These vesicles are transported in a MT-dependent manner and are stalled with colcemid or colchicine treatment which eventually prevented furrow invagination.[18,23,35] It is unclear if such a membrane reservoir is present at the *S. arctica* cortex where the furrows are first initiated. Brefeldin A treatment in *Drosophila* embryos inhibits furrow progression in the final stage of cellularization.[15,18]

On the dose series, a high dose of Brefeldin A at 3 µg/ml interferes with and in some coenocytes blocks cellularization, which establishes that Golgi-mediated trafficking is required for the process and is shown in Figure EV4A to Figure EV4C. All quantitative measurements are made at 1.5 µg/ml, a dose that leaves ingression intact, and the Methods now state this separation explicitly so that no measurement is read as belonging to the blocking dose.
On the resemblance to MTs depolymerization, the referee is right that this could not be concluded from the images, and we have therefore tested it. Furrow spacing variability was measured identically in the experiments. They are statistically indistinguishable, and so are their two controls, which is what makes the comparison meaningful.

Figure 4C: (C) Variability of furrow spacing within a coenocyte, from the same measurements. Methanol 0.212, BfA 0.433, $p = 0.0047$. Measured the same way in both experiments, the two perturbations are indistinguishable from one another (MBC 0.446 versus BfA 0.433, $p = 0.66$) and so are the two controls (DMSO 0.213 versus methanol 0.212, $p = 0.67$).

On what is learned from the experiment, the answer is that the two perturbations dissociate, and this is now the organising result of the manuscript. Ten measurements are compared between them in Figure 4F, each as a log2 fold change against its own control and grouped by the property it belongs to, and Figure 4G summarises which machinery contributes to which property.

Line 389-395: Nuclear positioning, by contrast, was not detectably affected as neither inter-nuclear distance nor its variability differed from controls (Figs. EV4E, F). Ingression rate (Fig. 4E), its variability (Fig. EV4G) and the maximum length reached by furrows (Figs. EV4H, EV4I) were likewise comparable to controls. Cellularization nonetheless took longer to complete (from 40 to 50 min, Fig. EV4J), and the cells released at the end were of more variable size (0.140 to 0.266, Fig. EV4K).

Trafficking is therefore required for cellularization to proceed, since the high dose blocks it, yet at a dose that leaves ingression rate untouched what it contributes is where furrows form and whether cellularization finishes on time. Nuclear organisation is unaffected at that dose, in Figure EV4E and Figure EV4F, which is precisely where the two perturbations part company and is the reason the resemblance in furrow positioning is informative rather than trivial.

The closing paragraph of this section has been rewritten as described in our response to comment 3, and it now ends on this comparison rather than on a general statement.

Significance
As I note in detail in the previous section, Here the authors extend this work to the ichthyosporean Sphaeroforma arctica, exploring the fascinating process by which a syncytial life stage cellularizes, and seeking to define the role of microtubules and Golgi trafficking. Their new expansion microscopy delivers impressive images of membrane and the cytoskeleton during this process and has the promise of answering important questions about the roles of microtubules and membrane trafficking. I thus went into my review quite excited. However, as I detail below, while some aspects of the process are carefully quantified, the current manuscript draws multiple broad conclusions that do not seem fully supported by the limited data provided. This substantially reduced my enthusiasm.

We are grateful that the referee finds the ExM compelling. The concern that broad conclusions outran the data has driven most of this revision, and every conclusion in the manuscript is now either carried by a measurement made across the dataset or has been removed.

Reviewer #2
Evidence, reproducibility and clarity
Summary

This interesting paper is a follow-up from Dudin et al.'s seminal 2019 eLife paper describing cellularization in the ichthyosporean Sphaeroforma arctica. In that earlier story, a role for microtubules (MTs) in cellularization was supported by treatment with the MT-depolymerizing drug MBC, which deeply affected nuclear spacing and the regularity of cellularization. However, the difficulty of imaging microtubules at the time had prevented more in-depth functional studies. This technical barrier has now been lifted by ultrastructural expansion microscopy (U-ExM), and this paper thus picks up where the earlier study left off.
The study combines live imaging, drug treatments, electron microscopy and ultrastructural electron microscopy to support a role for microtubules, nuclei, and membrane trafficking in sustaining the fidelity of S. arctica cellularization. An extensive live imaging dataset and careful image quantifications reinforce and expand the previously published observation that microtubules, while dispensable for cellularization to occur at all, are necessary for it to occur with proper timing and spacing. UEx-M images support the idea that actin and microtubules guide plasma membrane invaginations, and centrifugation experiments support a role for nuclear positioning in ensuring fidelity of cellularization. Finally, Brefeldin A treatment followed by live imaging and U-ExM supports a role for membrane trafficking in furrow positioning and elongation.

Major comments

The conclusions are adequately supported by the data, and their limitations are transparently acknowledged: notably, it is not fully clear by what mechanisms nuclei guide cellularization, and whether those mechanisms depend on microtubules or not. Below are a few points where I feel additional data (or better visualization, or additional verbal caveats) could improve the manuscript.

We are grateful that the referee reads the work as picking up where the 2019 study left off, and that they find the conclusions supported and the limitations transparently stated. Their four points are addressed below.

1) After 12,000rpm centrifugation, the authors point out that furrows "were misaligned, frequently enclosing multiple nuclei per compartment". This is not obvious in Figure 3C, where nuclei are only visible as empty spaces and only labelled (by asterisks) at a relatively early stage of furrow ingression. This point could be better supported by more explicit images (with nuclei more evident at late stages, even if only as blank spaces), or maybe by co-staining DNA and either membrane or F-actin in centrifuged samples.

The co-staining the referee suggests is now provided. Figure 3A and Figure EV3A show centrifuged coenocytes by U-ExM with DNA, membrane, actin and tubulin labelled together, so nuclei are seen directly rather than as gaps. Asterisks now mark nuclei throughout the time-lapse in Figure 3B, and Figure EV3D shows a centrifuged coenocyte at both low and high display contrast. As set out in our response to Ref 1-2, nuclei are scored in live imaging as regions from which FM4-64 is excluded.

2) In centrifuged samples (Figure 3A), some staining is visible in tubulin channel of the nuclei-free part, but does not correspond to discrete, observable microtubules. Could the authors comment on this? Do they think these represent diffuse, perhaps damaged microtubules (nucleated independently of nuclei?), or perhaps free tubulin, or mere background?

We have measured it rather than interpreted it. These objects are short tubulin-positive fragments, clearly distinct from nuclear asters in both number and length, and we report them separately for that reason. We do not claim to know whether they are remnants of displaced MTs or independently nucleated, and the manuscript says so by describing them rather than assigning them an origin.

Figure EV3C: In the nuclei-depleted cortex of the same coenocytes, tubulin was present only as short fragments, a median of three per coenocyte and 0.95 µm long (see Data EV1), which are different objects from an aster and are not tested against it.

3) Similarly: in centrifuged samples Fig. 3C, the nuclei-free part of the cell does seem to cellularize, albeit slower and forming smaller compartments than the nucleated part. This suggests that nuclei (and microtubules?) guide cellularization, but are perhaps not necessary for it. Could the authors comment?

We agree, and this is now quantified. In the nuclei-depleted region furrows are more widely and more variably spaced, reach shorter lengths and ingress more slowly than in the nuclei-enriched region of the same coenocyte, yet they form and progress. Nuclei therefore guide cellularization without being required for it, which is also what Referee 3 takes from the same experiment.

Line 308-317: Furrow spatial patterning was dramatically altered: in nuclear-depleted cortical regions, furrow initiations were more frequent and closely clustered but failed to progress deeply (Figs. 3B and EV3D). Among the furrows that did progress, spacing was more variable in the nuclear-free region than in the nuclear-enriched half of the same coenocyte. (Fig. 3E). Since furrow spacing is reliably quantifiable only for furrows that progress, the effective furrow spacing in this nuclear depleted region appeared wider and was abolished upon MBC treatment (Fig. EV3F). Taking into account the high number of furrow initials in the nuclear-depleted region (Figs. 3B and EV3D), and furrow separation in the region enriched with nuclei suggests a minimum furrow exclusion zone around individual nuclei and their MT networks.

4) The bottom row of Figure 3C presents a series of experiments combining MBC treatment with 12,000rpm centrifugation, but if I am not mistaken these are not discussed at all in the text. This is unfortunate, as it would be interesting to know what the authors want to conclude from these experiments (whose results are however not quantified, perhaps limiting their scope). In my view, one reason to be interested in this dataset is that it could in principle inform epistatic relationships between nuclei positioning and microtubules, that are otherwise left open: do these guide furrow ingression independently of each others (in which case their effects should be additive) or does one act purely through the other (in which case the combined treatment should not be worse than individual treatments)? In any case, I would suggest either commenting on these data explicitly (perhaps with additional quantification guiding interpretations) or removing them.

The referee is right that these data were not discussed, and they are now quantified and interpreted. As set out in Ref 1-4, combining centrifugation with MT depolymerization does not add to the effect of nuclear displacement but reverses it, so that the region depleted of nuclei carries narrower gaps rather than wider ones. This is the epistatic test the referee proposes, and it argues against two contributions acting independently and in parallel. The nuclear contribution appears to run through the MT network.

Minor comments

- Figure 1 contains two distinct panel H's.
- Typo p. 6: "actomyosing"
- Page 7: "longer MT extensions associated with deeper furrows during later stages (Figure 2D, arrowheads)" rather seems to refer to Figure 2B or C.
- Line and page numbers are missing

All corrected.

Significance

This paper will be of broad interest to readers interested in the evolution of multinucleated cells and cellularization, and the recurrent involvement of actin and microtubules in these processes (see notably recent papers by the Brugués lab). While it could appear incremental in an ichthyosporean-centric perspective (notably compared to the 2019 eLife paper that had anticipated some of the conclusions), its comparative implications gives it an additional scope in my view. Perhaps this is something the authors themselves could emphasize a bit more in their own conclusion. Finally, although most techniques had been established in earlier papers by the same authors, this study further confirms their robustness and the power of S. arctica as an emerging cell biology model - another nice touch.

We have followed this suggestion. The closing section now sets our results against cellularization in Drosophila and in chytrids. Also, it compares it against recent work showing that partitioning by MT asters is intrinsically unstable, so that losing its control broadens the distribution of compartment sizes rather than shifting it. That is the pattern we observe in our study. We have hinted at this in the discussion, so that the comparative implications the referee points to are stated rather than left to the reader.

Reviewer #3 (Evidence, reproducibility and clarity (Required)):

In their manuscript, Araújo et al. described their investigation into the cellularisation process in Sphaeroforma artica during the multicellular stage of its life cycle. The coenocytes are relatively small at 50 µm in diameter, but the authors developed expansion microscopy and used a new actin probe to provide a detailed description of the ingression of the membrane furrow between adjacent nuclei. They compared the formation of the furrow in the presence or absence of microtubules, as well as in conditions involving regularly spaced or clustered nuclei. They concluded that, while microtubules were not essential to the formation and ingression of the furrow, they were required for the fidelity of the process, as stated in the title of the manuscript.

A better quantification of the ingression pattern would have improved the data. The centrifugation experiment is particularly interesting, as it forces the accumulation of nuclei on one side of the coenocytes. Surprisingly, this only partially perturbs the position of the furrow. This showed, on the one hand, that nuclei contribute to the positioning of the furrow close to them, and, on the other hand, that an additional mechanism exists independently of them. The analysis does not clarify whether the absence of microtubules in these centrifuged states affects the position of the furrow. Figure 3C seems to suggest partial rescue (suggesting that the contribution of nuclei is microtubule-dependent, but that the peripheral membrane has its own partitioning mechanism), but this has not been quantified.

The quantification of ingression is considerably expanded, and the framework it now detailed in Ref 1 - 2.

On the centrifuged coenocytes lacking microtubules, this is now quantified. As set out in Ref 1 - 4, the relationship between nuclear position and furrow spacing does not merely weaken when microtubules are removed, it reverses, which supports their inference that the contribution of nuclei is microtubule-dependent. In the same experiment, the local slowing of ingression where nuclei are absent persists whether or not MTs are present, which is consistent with the second half of their reading, that the cortex retains a partitioning mechanism of its own.

One minor concern is that it remains unclear whether microtubule disruption affects the positioning of the nuclei, as stated in the text but not confirmed by the quantification (Figure 1F).

Figure 1F now reports the variability of inter-nuclear distance within each coenocyte, which roughly doubles upon microtubule depolymerization, while the mean is unchanged and is shown separately in Figure EV1G. The claim in the text is therefore about regularity rather than about mean spacing, and it is now matched by the panel that supports it. This is set out in full in Ref 1 - 2.

Reviewer #3 (Significance (Required)):

The work is primarily descriptive, and the processes determining the position of the furrow and driving its ingression remain unknown. Nevertheless, the work provides beautiful images of a poorly described yet potentially informative system. These are distant relatives of animals with interesting common characteristics, such as the cellularisation process. The conservation of this process may reveal some key fundamental properties of multicellularity. Despite its limited conceptual advances, the manuscript is thus innovative and interesting.

We thank the referee for their assessment. The revision strengthens what the paper claims. Comparing our two titratable perturbations across ten measurements shows that furrow positioning and furrow ingression dynamics can be disturbed independently of one another, and that neither prevents cellularization from completing (Figure 4F and Figure 4G). What is degraded is fidelity rather than execution of cellularization.

October 7, 2026

RE: Life Science Alliance Manuscript #LSA-2026-03917-T

Dr. Omaya Dudin
University of Geneva
Biochemistry
Geneva
Switzerland

Dear Dr. Dudin,

Thank you for submitting your revised manuscript entitled "Microtubules coordinate cellularization in a coenocytic relative of animals". We returned this to two of the original reviewers from Review Commons (Reviewer 2 was unavailable). As you will see, both reviewers recommend publication. We would be happy to publish your paper in Life Science Alliance pending final revisions necessary to meet our formatting guidelines, and any changes you wish to incorporate from the comments of Reviewer 1.

MANUSCRIPT ORGANIZATION AND FORMATTING:

To avoid unnecessary delays in the acceptance and publication of your paper, please read the following information carefully. Full guidelines are available on our Instructions for Authors page, https://www.life-science-alliance.org/authors

Submission of a paper that does not conform to Life Science Alliance guidelines will delay the acceptance of your manuscript.

-LSA allows supplementary figures, but no EV Figures; please update your callouts for the Supplementary Figures in the manuscript: Fig EV1A=Fig S1A; while supplementary figures use the system supplementary Fig S1; Same applies to the Movies.
-Please add ORCID ID for corresponding (and secondary corresponding) authors - you should have received instructions on how to do so.
-Please add a Summary Blurb/Alternate Abstract in our system.
-Please add a Category for your manuscript in our system.
-Please add the X and Bluesky handles of your host institute/organization, as well as your own, and/or one of the authors, in our system.
-It is recommended to exclude figures from the manuscript text and upload them separately.
-Please add the authors' contributions to the system as well.
-Please consult our manuscript preparation guidelines https://www.life-science-alliance.org/manuscript-prep and make sure your manuscript sections are in the correct order.
-Please add your main, supplementary figure, and movie legends to the main manuscript text after the references section.
-Please add a Conflict of Interest statement to your main manuscript text. If there is no COI, please use this formulation: "The authors declare that they have no conflict of interest."
-LSA permits merged Results and Discussion sections for short manuscripts. We invite you to consider separating these sections, but we leave this decision to you.
-Thank you for including the clear and rigorous statement on AI use!

We encourage our authors to provide original source data, particularly uncropped/-processed electrophoretic blots and spreadsheets for the main figures of the manuscript. If you would like to add source data, we would welcome one PDF/Excel-file per figure for this information. These files will be linked as supplementary "Source Data" files.

It is Life Science Alliance policy that if requested, original data images must be made available to the editors. Failure to provide original images upon request will result in unavoidable delays in publication. Please ensure that you have access to all original data images prior to final submission.

If you are planning a press release on your work, please inform us immediately to allow informing our production team and scheduling a release date.

We welcome submissions of potential cover images for the issue of LSA in which your work would appear. If you have high quality images associated with this work, please feel free to email these, with a caption, to the journal office.

LSA encourages authors to provide a 30-60 second video where the study is briefly explained. These videos will be appear embedded with the manuscript online at Life Science Alliance, and on social media to promote the published paper and authors

(for examples, see https://docs.google.com/document/d/1-UWCfbE4pGcDdcgzcmiuJl2XMBJnxKYeqRvLLrLSo8s/edit?usp=sharing). Corresponding or first-authors are welcome to submit the video. Please submit only one video per manuscript. The video can be emailed to contact@life-science-alliance.org

FINAL FILES:

To upload the final version of your manuscript, please log in to your account: https://lsa.msubmit.net/cgi-bin/main.plex
You will be guided to complete the submission of your revised manuscript and to fill in all necessary information. Please get in touch in case you do not know or remember your login name.

The following items are required for acceptance.

-- An editable version of the final text (.DOC or .DOCX) is needed for copyediting (no PDFs).

-- High-resolution figure, supplementary figure and video files uploaded as individual files: See our detailed guidelines for preparing your production-ready images, https://www.life-science-alliance.org/authors

-- Summary blurb (enter in submission system): A short text summarizing in a single sentence the study (max. 200 characters including spaces). This text is used in conjunction with the titles of papers, hence should be informative and complementary to the title. It should describe the context and significance of the findings for a general readership; it should be written in the present tense and refer to the work in the third person. Author names should not be mentioned.

The license to publish form must be signed before your manuscript can be sent to production. A link to the license to publish form will be available to the corresponding author only. Please take a moment to check your funder requirements.

Reviews, decision letters, and point-by-point responses associated with peer-review at Life Science Alliance will be published, alongside the manuscript. If you do want to opt out of having the reviewer reports and your point-by-point responses displayed, please let us know immediately.

Thank you for your attention to these final processing requirements. Please revise and format the manuscript and upload materials as soon as you are able.

Thank you for this interesting contribution to the literature. We look forward to publishing your paper in Life Science Alliance.

Sincerely,

Tim Fessenden
Scientific Editor
Life Science Alliance

Reviewer #1 (Comments to the Authors (Required)):

As I noted in my initial review, the last several years have seen major advances in our understanding of the basic cell biology of the set of single cell animal relatives, led by the authors and their colleagues. These groups have developed several as models and pioneered remarkable microscopy approaches to examine their cell biology. Here the authors extend this work to the ichthyosporean Sphaeroforma arctica, exploring the fascinating process by which a syncytial life stage cellularizes, and seeking to define the role of microtubules and Golgi trafficking. Their new expansion microscopy delivers exciting images of membrane and the cytoskeleton during this process. The original version had issues that undermined its impact. The authors have done an exceptionally impressive job of addressing all of my concerns, clarifying and tempering text and adding substantial quantification. This work answers important questions about the roles of microtubules and membrane trafficking in this divergent animal relative and will be of broad interest to cell, developmental and evolutionary biologists.

I had a few very minor suggestions for the authors as they prepare the final version, none of which require re-review.

Fig 1J Perhaps make the orange lines a bit more obvious

Fig 3B Indicate which end is "nuclei enriched" a(as they do in EV3 which is lovely by the wat) and point out using arrows an example of "In nuclear-enriched regions, furrows were misaligned, frequently enclosing multiple nuclei per compartment" . I think I understood this from the phenotypic descriptions but making it explicit on the figure would be helpful

Fig 3D. The authors state "This advantage in nuclear-enriched regions was lost upon MBC treatment" but this panel suggests

this is an overstatement.

Reviewer #3 (Comments to the Authors (Required)):

The additionnal quantifications have properly addressed my minor concerns.

October 12, 2026

RE: Life Science Alliance Manuscript #LSA-2026-03917-TR

Prof. Omaya Dudin
University of Geneva
Biochemistry
Geneva
Switzerland

Dear Dr. Dudin,

Thank you for submitting your Research Article entitled "Microtubules coordinate cellularization in a coenocytic relative of animals" to LSA, revised according to our journal formatting requirements. It is a pleasure to let you know that your manuscript is now accepted for publication. Congratulations on this interesting and beautifully documented work.

The final published version of your manuscript will be deposited by us to PubMed Central upon publication. Your article will publish open access upon publication under a CC-BY license.

Your manuscript will now progress through copyediting and proofing. It is journal policy that authors provide original data upon request.

Reviews, decision letters, and point-by-point responses associated with peer-review at Life Science Alliance will be published, alongside the manuscript. If you want to opt out of having the reviewer reports and your point-by-point responses displayed, please let us know immediately.

IMPORTANT: If you will be unreachable at any time, please provide us with the email address of an alternate author. Failure to respond to routine queries may lead to unavoidable delays in publication.

Scheduling details will be available from our production department. You will receive proofs shortly before the publication date. Only essential corrections can be made at the proof stage so if there are any minor final changes you wish to make to the manuscript, please let the journal office know now.

DISTRIBUTION OF MATERIALS:
Authors are required to distribute freely any materials used in experiments published in Life Science Alliance. Authors are encouraged to deposit materials used in their studies to the appropriate repositories for distribution to researchers.

You can contact the journal office with any questions, contact@life-science-alliance.org

Again, congratulations on a very nice paper. I hope you found the editorial process to be constructive and are pleased with how the manuscript was handled overall. We look forward to future exciting submissions from your lab.

Sincerely,

Tim Fessenden
Scientific Editor
Life Science Alliance
